# International market exposure to sovereign ESG

**DOI:** 10.1080/20430795.2022.2148817

**Published:** 2022-11-25

**Authors:** Christian Morgenstern, Guillaume Coqueret, James Kelly

**Affiliations:** a MRC Centre for Global Infectious Disease Analysis, Imperial College London, Norfolk Place, London, UK; b EMLYON Business School, Ecully, France; c Connected Asset Management, London, UK

**Keywords:** Factor demand, longitudinal models, sovereign ESG, sustainable investing, G11, H11, Q59

## Abstract

We quantify equity and bond market sensitivity to sovereign ESG scores and their variations which, theoretically, is equivalent to evaluating the demand for ESG at the global scale. We do so by estimating a longitudinal model, at the issue level, that captures exposures to sovereign ESG factors for both equity and fixed income indices. In spite of the surging interest in ESG investing, our results do not support a strong impact of ESG factors on the returns of international markets, implying that the demand for ESG at the country level is not a significant driver of prices. Nevertheless, we document a strong association between GDP growth and ESG scores at the country level.

## 1. Introduction

Sustainable investing has experienced a recent surge and a large majority of institutional investors now integrate non-financial criteria in their decision process, in particular Environmental, Social and Governance (ESG) factors, and green investments are gaining traction among retail investors. This has been widely documented in the literature (Coqueret 2022).^1^

One focal question in the field pertains to the relationship between sustainability and financial performance, i.e. the extent to which an ESG investor would have to abandon profitability when buying socially responsible assets (see, Chapter 4 of Coqueret 2022, for equities and Flammer 2021, for bonds). Given the numbers of degrees of freedom in studies dedicated to this issue, it is not surprising that conclusions differ from one contribution to another. Meta analyses such as Kim (2019) and Whelan, Atz, and Holt (2021) confirm this lack of robustness, and non-linearities (Barnett and Salomon 2006; Brammer and Millington 2008; Harjoto, Jo, and Kim 2017) may be one cause thereof.

Lately, scholars in the social sciences have focused on the reasons that drive investors toward ESG assets. This is important because in markets that are not very elastic, net demands are likely to drive returns and performance (Gabaix and Koijen 2022). Appetite for ESG might come from financial motivations (e.g. sustainable firms are less risky (Becchetti et al. 2015; Gonçalves, Pimentel, and Gaio 2021; Hoepner et al. 2021) and are more resilient,^2^ typically because they are less exposed to transition and physical risks) or for altruistic purposes (the so-called ‘*warm glow*’ effect, see Mahmoud 2020).

In this paper, in contrast to traditional studies that focus on firm-level scores, we are interested in the impact of *countries'* ESG ratings on their own financial markets, considering both equity and fixed income markets. To do so, we resort to standard panel models that seek to capture these markets' exposure to sovereign ESG scores. These scores are computed as synthetic averages of metrics disclosed by major international institutions, such as the World Bank or the United Nations. The study encompasses more than 70 countries in total over the period 2000–2021.

A key aspect of the model is that we include variations in scores (also known as ESG momentum). This specification, while also making sense theoretically (momentum apprehends ESG dynamics), has a convenient interpretation for the demand for ESG in financial markets. The estimated coefficients related to ESG momentum are theoretically linked to the net demand for the corresponding variable. This is interesting because it provides another angle to the topic of ESG demand. There are currently two ways to study the appetite for sustainable assets. The first one is to carry out surveys, especially directed to professionals in the field (asset owners and managers). This is the path chosen by Van Duuren, Plantinga, and Scholtens (2016), Eccles, Kastrapeli, and Potter (2017) and Lagerkvist et al. (2020). The second route is to analyse flows directly, as in Hoepner and Schopohl (2018) and van der Beck (2021). The latter reference seeks to explain the high returns of ESG funds in recent years. One drawback of these two approaches is their scope, which is by construction limited, either in investor types, geographical areas, or cross-sectional coverage. Surveys often reach a few hundred individuals and flows are generally scrutinized for a few dozen institutional investors at most.

We run our models on several sample sizes and at different levels of issue granularity. Our central results do not support the claim that market fluctuations reflect strong demand in ESG. Across all our model configurations, coefficients pertaining to ESG variables fluctuate around zero and are associated with test statistics that are rarely larger than two in magnitude, which underlines their statistical weakness. Conversely, latent demands, which correspond to the part of returns that cannot be explained by ESG predictors, are sizeable. Our results are in line with those of Berk and Binsbergen (2021) who suggest that the proportion of ESG investors is still too low to have a marked impact on financial markets.

The remainder of the paper is structured as follows: The literature review is located in Section 2, followed by a discussion on the demand model itself in Section 3. Section 4 is dedicated to estimations, and we provide numerous results on the calibration of the demand model, along with various parametrisation options. In Section 5, we list potential applications of our findings for practitioners. Section 6 concludes. Figures dedicated to robustness checks are available in the Appendix.

## 2. Literature review

Our paper relates to two streams of literature. First, it belongs to the contributions that work with sovereign sustainability data, often in connection with its link to economic or financial performance. Sovereign ESG metrics are consistent across rating agencies: Bouyé and Menville (2020) document high correlations among sovereign sustainability scores across countries (as reported by Standard & Poors, Moody's and Fitch). This stands in contrast with firm-specific ESG scores (see Berg, Kölbel, and Rigobon 2020; Dimson, Marsh, and Staunton 2020 and Abhayawansa and Tyagi 2021).

The international agencies can then use these ratings to adjust their risk assessments, a topic studied by Angelova et al. (2021). Cevik and Jalles (2022a, 2022b) run similar analyses for sovereign default risk. In particular, Capelle-Blancard et al. (2019) and Crifo, Diaye, and Oueghlissi (2017) find that high ESG countries have both lower credit risk and thus lower bond spreads and lower borrowing costs. Semet, Roncalli, and Stagnol (2021) document that the E and G pillars are the most salient in explaining the differences in sovereign bond yields, while Rahman et al. (2021) and Pineau, Le, and Estran (2022) contend that the relative importance of sustainability in credit ratings varies between emerging and developed countries.

Outside fixed income markets, C. Chang et al. (2020) studies the impact of countries' CO2 emissions on their relative stock markets. Their Granger causality tests suggest that stock markets cause emissions, but not the other way around. Morgenstern, Coqueret, and Kelly (2021) exploit sovereign ESG indicators in order to build sustainable trend-following strategies. Similarly, Cheema-Fox, Serafeim, and Wang (2021) link countries' vulnerabilities to climate change and exploit them in trading strategies on foreign exchanges. In Y. Chang, He, and Mi (2021), the authors investigate corporate payout policies (e.g. dividends and share repurchases) in relation to the firms' countries' exposure to climate risk. Finally, another insightful study is that of Zhang, Zhao, and Lau (2022), who report that sovereign ESG is linked to corporate investment decisions. At the macro-economic level, both Vărzaru, Bocean, and Nicolescu (2021) and Diaye, Ho, and Oueghlissi (2022) document the positive effect of sustainability on GDP per capita and GDP growth. Banking flows across countries, is investigated by Avci and Esen (2021) in relationship to macroeconomic sustainability.

A second recent stream of literature pertains to the estimation of demand for particular characteristics of assets. The seminal contribution is that of Koijen and Yogo (2019), in which an equilibrium model with heterogeneous agents is solved to obtain prices observed on markets. The importance of agents (e.g. their assets under management) is known, as well as assets' characteristics. A fixed-point method is used to derive the demand of each agent in all characteristics, in addition to a latent demand which explains the portion of prices that are not captured by the characteristics (often, a large portion). The approach of Koijen and Yogo (2019) has been used to evaluate investor-specific (granular) demands by Noh and Oh (2021) and van der Beck (2021). Both contributions aim to estimate the investors' appetite for green/ESG assets. The latter one shows that the recent rally around sustainable stocks was caused by a substantial increase in demand and is the main reason why they have enjoyed above average returns in the recent period. This is in line with recent models that underline the importance of aggregate demand in explaining price movements (Gabaix and Koijen 2022).

We end this section by recalling some theoretical contributions in sustainability-based asset pricing. The seminal paper of Pástor, Stambaugh, and Taylor (2021) argues that ESG should be costly but the more complex version (Avramov et al. 2022) contends that this link may be reversed where there is high rating uncertainty. This last study was intended for firm-specific uncertainty in ESG scores, whereas in the sovereign data space, ambiguity is much less an issue.

## 3. The model and its relation with net aggregate demand

### 3.1. Theory

The standard approach to estimate the sensitivity of aggregate markets to the corresponding ESG scores is to run a panel model which is motivated by the fact that we can benefit from the heterogeneity between countries in our data set.

$$rt+1,n=at+1,n+\sum_{k=1}^{K}b\left(k\right)s_{t,n}^{\left(k\right)}+et+1,n,\;$$

where rt+1,n is the one period ahead (t+1) return of market *n* (say, the United States) and $s_{t,n}^{\left(k\right)}$ is a score that captures the kth dimension of sustainability (or, possibly some control variable that might be included in the model). b(k) is the coefficient that captures the link, while at+1,n is a possible country-specific and/or time effect. Fundamentally, the above equation is a particular case of the more general model proposed in Gu, Kelly, and Xiu (2020) in which future returns are represented as a nonlinear mapping rt+1,n=g(xt,n)+et+1,n, where xt,n stacks the information available at time *t* for asset *n*. Therefore, in the case of our baseline model, we only consider sustainability-based criteria, combined with a *linear* panel. We are interested in inference and not prediction, and inference is much simpler with linear models, for which test statistics are well understood.

However, we additionally propose a small modification of the above specification based on the theoretical results of Coqueret (2021), which we briefly recall below. This allows a convenient equilibrium-based link between estimates and aggregate demands. Therein, the assets are simple stocks and the attributes are standard characteristics analysed in the asset pricing literature (capitalization, accounting ratios, etc.). In this paper, assets will be international indices representing the two major asset classes (equities and fixed income) and their *K* attributes (detailed below in Section 3.2) will be the ESG dimensions of the countries from which they originate. We write $c_{t,n}^{\left(k\right)}$ for these characteristics ^3^ where again *t* is the time index, *n* the country index and *k* the attribute index.

One assumption in Coqueret (2021) is that some agents on the market have linear demands in these characteristics, plus two additional terms. The first term is the logarithm of the price of the asset, which, akin to the demand curve in economics, will determine the sensitivity of investors to the price of the asset. The second term is simply an error term which corresponds to the demand that is unrelated to the characteristics. In addition, other traders act as market makers and provide supply that is also orthogonal to the characteristics. The natural consequence is that, upon market clearing, logarithmic prices are also linear in the characteristics of the indices.

This analytical form is convenient, because, when taking log-returns, we obtain expressions that are linear both in the characteristics and in their changes in time: $\Delta c_{t,n}^{\left(k\right)}=c_{t,n}^{\left(k\right)}-c_{t-1,n}^{\left(k\right)}$. Formally, the expression reads:

(1) $$rt+1,n=\alpha t+1,n+\sum_{k=1}^{K}\left(\beta_{t+1}^{\left(k\right)}c_{t,n}^{\left(k\right)}+\eta_{t}^{\left(k\right)}\Delta c_{t,n}^{\left(k\right)}\right)+\epsilon t+1,n,\;$$

and the coefficients $\beta_{t+1}^{\left(k\right)}$ and $\eta_{t}^{\left(k\right)}$ can be viewed as changes in scaled demands for the characteristics and scaled demands, respectively. In this paper, we are interested in the latter.

Let us briefly explain how the model works in a simple example with only one characteristic ct (say, carbon emissions) and one country. In this case, the price is written pt=edtct−1+et, where et is some error term that aggregates the demand that is not driven by past emissions (likely, the majority of it). Note that decisions are based on past values, hence the time lag in ct−1. dt is a scaled demand, where the scaling is intended for the quantities to make sense. It is important to note that the scaling would be the same for all attributes if there were many. The demand dt will be useful for two purposes:

- its *sign* (do agents *buy* or *sell* more indices from countries with high/low emissions) and
- its *magnitude*, with respect to other attributes (e.g. when comparing the relative importance of emissions versus freedom of the press, or versus control of corruption).

The log-return is thus:

$$\begin{array}{rl} rt+1=\log(pt+1/pt) & =dt+1ct+et+1-dtct-1-et\\ & =(dt+1-dt)ct+dt(ct-ct-1)+\;\mathrm{innovation}\;\mathrm{term}\\ & =\Delta dt+1ct+dt\Delta ct+\;\mathrm{innovation}\;\mathrm{term},\; \end{array}$$

which is the simplest form of Equation (1). Therefore, if returns (on the left) are known, as well as characteristics (ct, on the right), the demand terms are the only unknowns. In the particular case of one asset (one country), they can be estimated via a simple regression. If many countries are considered, a panel approach is the standard way to proceed.

We underline that the regression specification in Equation (1) is relatively mainstream in the asset pricing literature, except for the difference (Δ) terms (see, e.g. Fama and French 2020). The coefficients measure the sensitivity of future returns to characteristics, or variations thereof. For instance, the demands in our economic model are the exposures of future returns to changes in the underlying characteristics, after controlling for the levels of these characteristics.

The analysis will be carried out for two types of investors separately: equity investors on one side and fixed income investors on the other. The rationale for this choice is that both market types behave very differently and agents that operate on them have different mindsets and objectives.

### 3.2. Sovereign sustainability scores

In this section, we detail the construction of the sovereign ESG scores, which we will use to analyse the demand for sustainability. We follow the same approach as outlined in Morgenstern, Coqueret, and Kelly (2021). This paper constructed *broad* ESG metrics, but only focused on a fraction of the liquid markets. The aim was to improve the ESG footprint of a trend-following trading scheme.

The construction of the sovereign ESG scores follows the same model as firm-specific scores. The three pillars (Environmental (E), Social (S) and Governance (G)) are split into issues which in turn may be linear combinations of sub-issues. We provide an overview of the pillars, issues and sub-issues used in our paper in Table B.2 in the Appendix.

**Figure 1.**
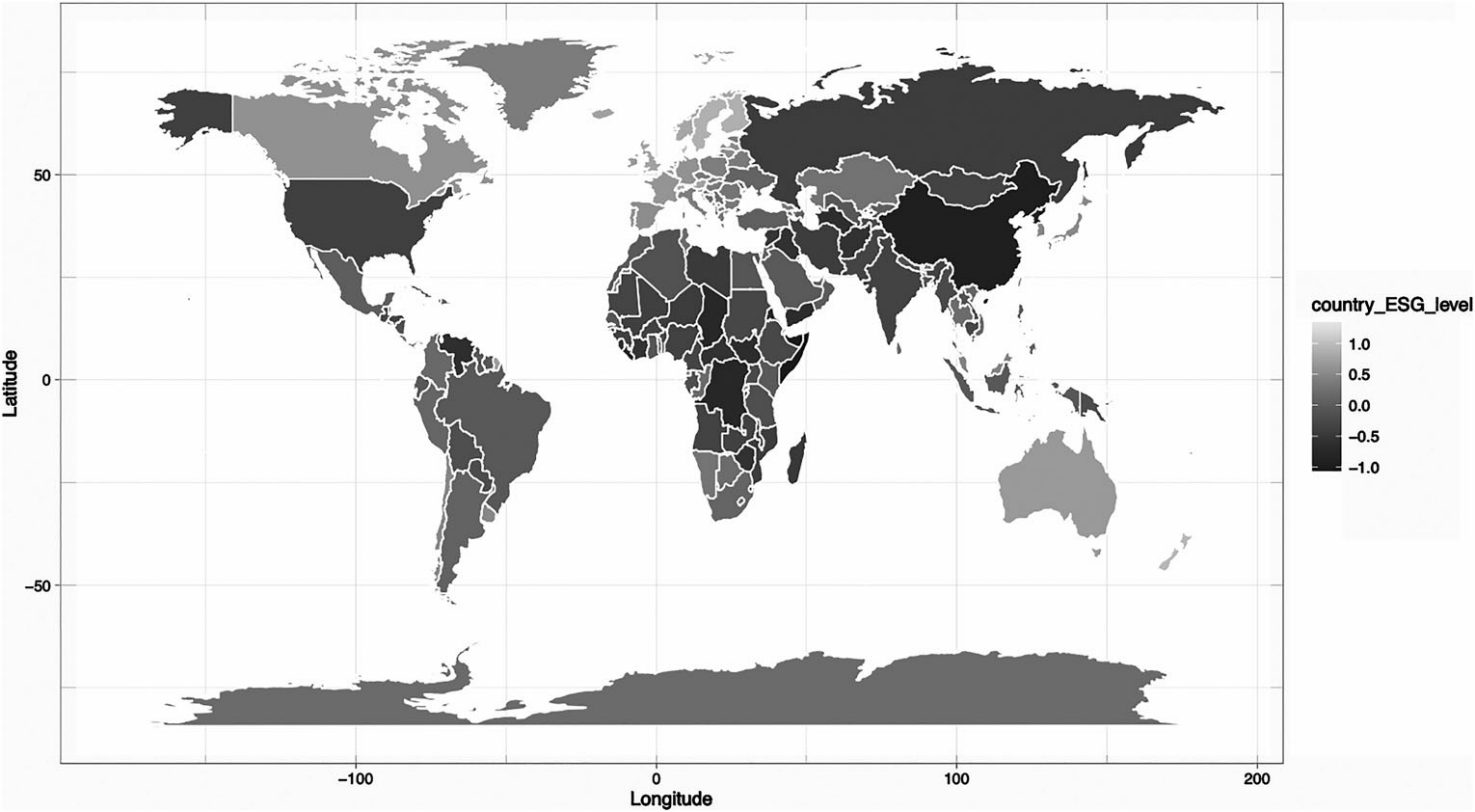
Heatmap of Country ESG scores.

Most of the underlying data is provided by supranational institutions such as the IMF, the World Bank, or independent charitable organizations. ^4^ This is one of the main differences with firm-level ESG scores for which the data acquisition step is much more challenging as there is a lack of standardization between companies providing the underlying data. This is the main reason why we see less dispersion in sovereign ESG scores, in comparison to corporate ESG metrics (Bouyé and Menville 2020; Berg, Kölbel, and Rigobon 2020; Dimson, Marsh, and Staunton 2020; Abhayawansa and Tyagi 2021). The data is provided on an annual basis. Figure 1 provides a map of the aggregate ESG scores across all countries and all dates for which data is available.

Our construction of a Macro ESG model is based on a wide range of diverse data sets. Other approaches are possible, most notably the UN Sustainable Development Goals, ^5^ which have gained in popularity over the last few years.

### 3.3. Properties of macro ESG scores

The data is sampled at the annual frequency and we use it to construct the scores as follows:

- We compute z-scores (normalized across countries) for sub-issues (or issues if there are no sub-issues);
- We combine the scores at the issue level. The issues are equally weighted and aggregated into each pillar (E, S & G) which are finally averaged into the ESG score at the country level. This is done for each date and country, and at all the levels. The aggregations are performed using equal weighting at all steps of the process.

**Figure 2.**
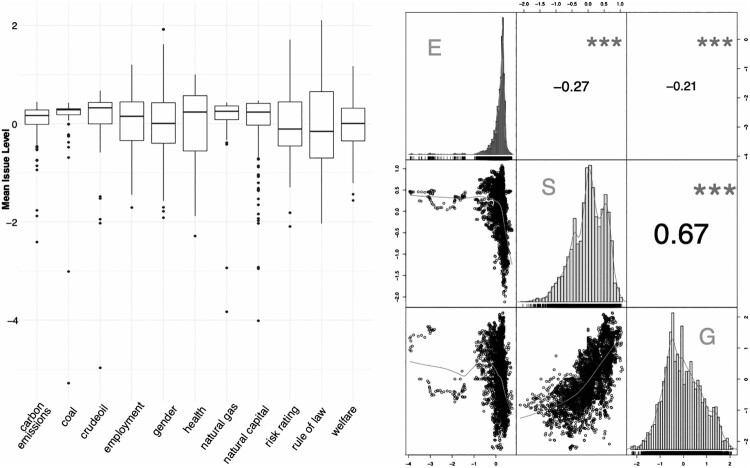
Descriptive plots of the ESG scores. Notes: The panel on the left shows a boxplot of scores at the issue level. The panel on the right shows histograms, scatterplots and correlations of E, S & G pillar values across countries and years.

Data is collected for 254 countries since 1980. From 1995 onwards, we have high coverage across all sub-issues and countries. In fact, we are able to collect ESG sub-issue data for a longer period than market data, especially for emerging markets. We review the financial data used in the next section. In Figure 2, we display some key statistics of the ESG data at the pillar and issue level.

### 3.4. Financial data

The financial data we work with are aggregate indices and the markets under consideration are summarised in Table B.3 in the Appendix. We use indices as assets because they provide a much larger universe than traded future contracts. ^6^

For equity markets, we use MSCI indices, which provide a good chronological coverage across continents. For most developed markets, the coverage starts in the 1970s but for emerging markets, the data is restricted to more recent dates. For fixed income markets, we use Bloomberg indices as they provide a wide range of markets and good geographical coverage. ^7^

## 4. Estimations

We investigate the various aspects of model choice, both in terms of parametrisation and estimation. Our main results are included in this section, with additional plots for robustness checks in the Appendix.

### 4.1. Baseline estimates and macro-economic controls

Our baseline results pertain to a model with fixed effects ^8^ meaning that the constant in Equation (1) is estimated at the country level. We proceed with rolling windows, so that, each quarter, we estimate the coefficients for each pillar (E, S and G), given the sample of the 10 previous years (40 observations per country). The choice of the rolling sample size is the results of a tradeoff that can be explained as follows. First, we seek to measure *local* demand (chronologically), so that we need samples that are not too long - this will allow to track the dynamics in loadings. But, at the same time, 2–4 years may be too short, especially when dealing with many predictors (e.g. at the issue level, see below). For the sake of completeness, results for 5 and 20 year samples will be provided in the Appendix in Figure C.9.

To explain this result, we note that sovereign ESG data is typically available on an annual basis and tends to move relatively slowly. The infrequent updating of the data is due to the fact that the process of change at the sovereign level takes more time, compared to the individual company level. Typically, for sovereign nations, this involves: a public and/or policy debate; a translation of that policy debate into legislation; the passing of this legislation through parliaments; an implementation phase and ultimately improvements to the measured metrics. The scale of these changes is measured in years or decades rather than months.

**Figure 3.**
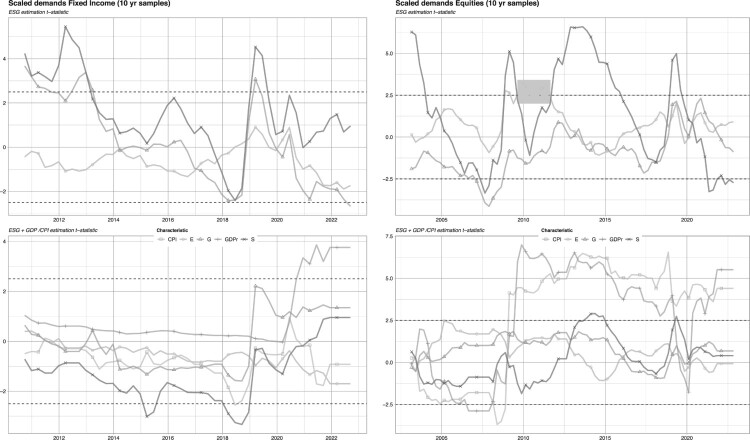
Baseline estimates and macroeconomic controls. Notes: We plot the time-series of *t*-statistics in fixed-effect panel models (Equation (1)) run on 10 years of data, at the *pillar level*. The panel on the left shows the analysis for fixed income markets while the one on the right pertains to equity markets. The horizontal dashed lines mark the 99% threshold for the significance of the coefficients. The bottom set of plots include macroeconomic controls as defined in model (2).

The time-series of the resulting *t*-statistics are depicted in the upper panels of Figure 3. We plot the *t*-statistics in place of coefficients for scale purposes and because the comparison with known benchmarks of significance (e.g. |t|>2.5 for the 99% level) is eased with *t*-statistics.

In the upper left panel of Figure 3, we see that demand for the S and G pillar is mostly positive in fixed-income markets, with short runs of negative signs in 2017–2018 and 2020–2021. On the contrary, the E pillar has mostly a negative demand. In equity markets, values are mostly positive for the E and S pillars, while often negative for the governance indicators, except towards the end of the sample. Overall, however, estimates are not particularly significant, with the exception of the social pillar, which has some short periods with strong coefficients.

In our baseline model, we attempt to measure the demand for sovereign sustainability using ESG factors only. However, investors do not invest in securities at the sovereign level solely for ESG motives, as they also demand financial performance. At the macro-economic level, key drivers for these investment decisions are economic variables which determine the economic outlook across countries. Investors aim to be long (*resp*., short) countries with good (*resp.*, poor) economic perspectives. To take this into account, we introduce changes in GDP, to control for economic growth, and changes in CPI, to control for inflation. These variables are scrutinized by investors, especially in fixed income markets, but also for global asset allocation (see Campbell and Viceira 2001; Vassalou 2003; Balduzzi and Moneta 2017).

This can be incorporated into our model as follows:

(2) $$rt+1,n=\alpha t+1,n+\sum_{k=1}^{K}\left(\beta_{t+1}^{\left(k\right)}c_{t,n}^{\left(k\right)}+\eta_{t}^{\left(k\right)}\Delta c_{t,n}^{\left(k\right)}\right)+\sum_{l=1}^{L}\psi_{t+1}^{\left(l\right)}\Delta m_{t,n}^{\left(l\right)}+\epsilon t+1,n,\;$$

where $\Delta m_{t,n}^{\left(l\right)}$ is the change in the lth macro variable for country *n* and time *t*.

In the lower panels of Figure 3, we plot the *t*-statistics of our baseline variables, along those of the macro-economic controls. We observe that the inclusion of macro variables can have a sizeable impact on the original results. First, significance levels are now barely reached any time at all for the ESG predictors. In addition, some signs may also change in the second specification.

An alternative model we propose is given by Equations (3) and (4) below. It consists of a two stage estimation, where, in the first step, we regress the asset market returns onto the corresponding changes in macro-economic variables. In the second stage, we use the residual (of the returns) from the first regression to estimate our sovereign ESG demand model.

(3) $$\mathrm{step}\;1:\;rt+1,n=\phi t+1,n+\sum_{l=1}^{L}\psi_{t+1}^{\left(l\right)}\Delta m_{t,n}^{\left(l\right)}+\nu t+1,n$$

(4) $$\mathrm{step}\;2:\;\nu t+1,n=\alpha t+1,n+\sum_{k=1}^{K}\left(\beta_{t+1}^{\left(k\right)}c_{t,n}^{\left(k\right)}+\eta_{t}^{\left(k\right)}\Delta c_{t,n}^{\left(k\right)}\right)+\epsilon t+1,n$$

One appealing feature of the two stage model is that, in theory, the first stage produces a set of clean residual returns which we can use for our analysis. This however comes at the cost of lower interpretability. We run this model comparison for equity markets in Figure C.8 in the Appendix. By and large, both configurations produce relatively similar results and our qualitative conclusions are not altered.

### 4.2. Issue level models

The analysis at the pillar level can be further refined to the issue level. Technically, scores are first built at the most granular level and then aggregated into issues, and then pillars. In order to stick to the ESG convention, we have ordered each issue such that a low (*resp.* high) value is less (*more*) sustainable. For example, and counter-intuitively, a high score on carbon emissions is in fact better for the environment due to this inverting the raw measurement. Table B.2 has the full list of details.

**Figure 4.**
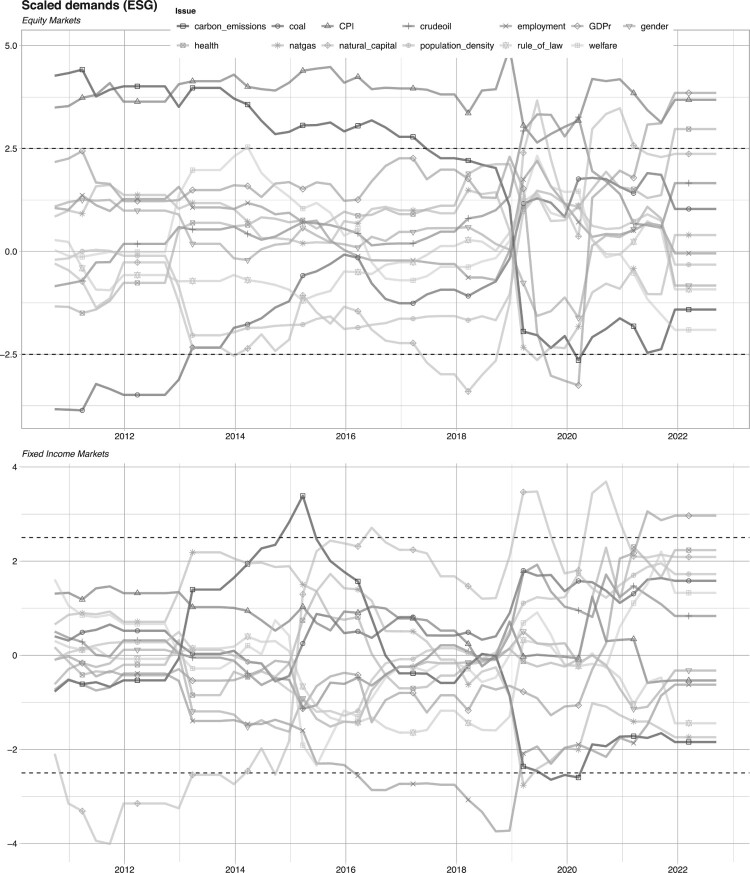
Analysis at issue level for equity & fixed income markets with macro variables. Notes: We plot the time-series of *t*-statistics in fixed-effect panel models (Equation (2)) estimated on 10 years of data, at the *issue level*. The panel on the top shows the analysis for equity markets while the one on the bottom pertains to fixed income markets. The horizontal dashed lines mark the 99% threshold for the significance of the coefficients.

In Figure 4, we plot the estimates when using issue predictors, as well as the macro-economic controls. These plots lead to several conclusions. First, the demand for sustainability factors at the issue level differs substantially for equity and fixed income markets, which is consistent with our conclusions for demands at the pillar level.

Second, we observe different demand dynamics for issues within a pillar. No issue is persistently and significantly in demand, with the exception of demand for *low* carbon emissions for equity markets during the period between 2012 and 2017. This is consistent with the recent increased focus on clean energy and energy transition, though it is unclear why the demand shrinks after 2017.

**Figure 5.**
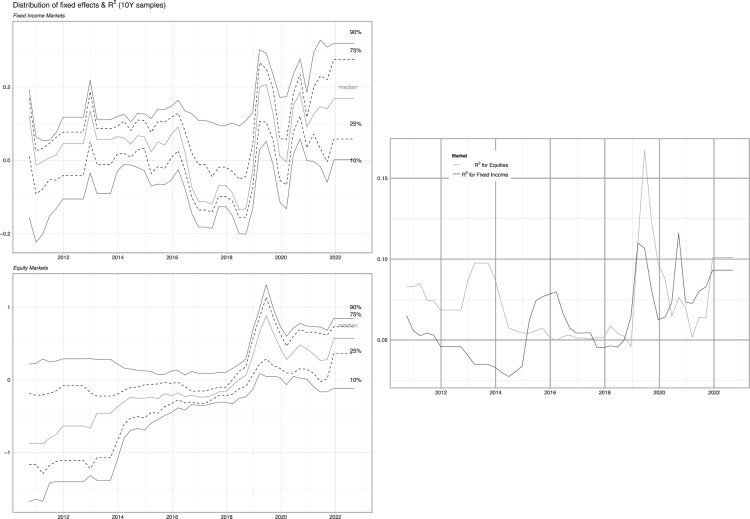
Latent demand distribution & R2 for equity & fixed income markets with macro variables. Notes: We plot the quantiles of the fixed effects in the panel models (Equation (2)) estimated on 10 years of data, at the *issue level*. The left hand top plot pertains to fixed income markets, the left hand bottom plot relates to equity markets and the right hand plot displays the R2 of the models over time.

In addition, in Figure 5, we provide the distribution of the fixed effects of the model we have estimated. Roughly speaking, we can interpret the average fixed effect as the country-specific intercept of a regression, i.e. the average return that is not explained by the independent variables (upon estimation). In Koijen and Yogo (2019), this is also referred to as *latent demands*. We note that the distribution of these (scaled) demands shifts to the left (or upwards, in the graph) over the course of our sample. This is due to the fact that E[rt|ΔGDP=0]≪0, that is the expected return is significantly negative given zero GDP growth. The upwards shift can be explained by increasingly lower growth expectations ^9^ and hence smaller negative expected returns given no GDP growth.

We evaluate, in Figure C.12 in the Appendix, the fixed effects for the model without macro-economic variables, and we observe a distribution of fixed effects which is more centred around zero (as GDP growth is not included), but also more dispersed. Again, this underlines the importance of the non-sustainable variables in the models. Typically, the market crash pertaining to the COVID-19 pandemic is absorbed in Figure 5 (with GDP and inflation), but not in Figure C.12 (without). This is confirmed with the R2 values, which are substantially higher in the former, compared to the latter.

### 4.3. Country selection

Heterogeneity between countries is an important component of the model, and estimates for individual countries are not expected to produce reliable results due to the sparsity of data. However, a reasonable question is to consider different country groupings to explore both the stability of our results as well as any informative patterns which may arise. For this country groupings need to have an intuitive meaning - we use the following sub-groups of countries: European countries, MSCI Developed markets and MSCI Emerging markets (a complete list for each is provided in Appendix B).

We find that our conclusions are robust to the selection of country groupings and we provide these in Figures C.5–C.7, in the Appendix. Unsurprisingly, we find a slightly higher significance of ESG factors in Europe relative to Developed markets or Emerging markets, which is consistent with the higher attention to ESG themes in Europe relative to other regions.

### 4.4. Association between changes in GDP and ESG scores

We concluded in the previous section that changes in GDP and CPI can be potent drivers of returns in these markets, which was not the case of ESG-linked predictors. In this subsection, we investigate a reverse model that sets the macro-economic variable to the left of the equation. Our aim is to understand the association of the macro variable and ESG proxies, contemporaneously. To this purpose, we focus on changes in GDP. We outline this model in Equation (5), where k=1,…,K spans ESG variables at the issue level. We resort to an estimation with fixed effects and rolling windows of 10 years.

(5) $$\Delta \mathrm{GD}Pt+1,n=\alpha t+1,n+\sum_{k=1}^{K}\left(\beta_{t+1}^{\left(k\right)}c_{t,n}^{\left(k\right)}+\eta_{t}^{\left(k\right)}\Delta c_{t,n}^{\left(k\right)}\right)+\epsilon t+1,n$$

We provide the time-series of *t*-statistics for this model in Figure C.14 in the Appendix. We note that no ESG factor dominates over the whole sample, and that *t*-statistics remain below the 1% significance level. Again, these somewhat underwhelming results corroborate our baseline conclusions on the relatively marginal importance of sovereign ESG for financial markets.

In Figure C.15, in the Appendix, we plot the fixed effects versus the ESG scores for each country. The panel is divided into the two time periods, and we provide results for the overall ESG scores and at the pillar level. The plots also include the regression lines for the data. During the early period of 2010–2018, we see only a moderate deviation from the median fixed effect (i.e. irrespective of the ESG characteristics) at the issue level. There is little to no differentiation of changes in GDP versus the level of the overall ESG score or the individual pillars scores. However, over the last few years we see an increasing deviation and a positive slope, except for the E pillar.

The regression of ΔGDP fixed effects versus the ESG scores is performed via the robust methodology of *M* estimators (see Huber 1981). Table 1 summarises the regression results. ^10^ From these, we can see that during the first period of 2010–2018 the contemporaneous association between the ΔGDP fixed effects and ESG scores have been very small indeed. Although the estimate of the coefficient of the E pillar is significant, it is quite small and, in fact, the intercepts are the most important terms.

**Table 1.** Regression results for ΔGDP fixed effects vs ESG scores.

Pillar	Term	Estimate	Std. Error	*t*-statistic	*p*-value	Confidence interval
*2010–2018*						
ESG	(Intercept)	0.0336	0.0018	18.8313		[0.030,0.037]
ESG	esg_score	0.0061∗	0.0033	1.8313	0.0672	[−0.000,0.013]
E	(Intercept)	0.0357	0.0015	24.0030		[0.033,0.039]
E	esg_score	0.0062∗∗∗	0.0021	2.9250	0.0032	[0.002,0.010]
G	(Intercept)	0.0354	0.0018	19.9048		[0.032,0.039]
G	esg_score	−0.0001	0.0018	−0.0766	0.9391	[−0.004,0.003]
S	(Intercept)	0.0336	0.0022	15.3171		[0.029,0.038]
S	esg_score	0.0046	0.0043	1.0768	0.2880	[−0.004,0.013]
*2019–2021*						
ESG	(Intercept)	0.1441	0.0071	20.3688		[0.130,0.158]
ESG	esg_score	0.0448∗∗∗	0.0135	3.3092	0.0009	[0.018,0.071]
E	(Intercept)	0.1575	0.0058	27.0910		[0.146,0.169]
E	esg_score	−0.0051	0.0082	−0.6182	0.5288	[−0.021,0.011]
G	(Intercept)	0.1405	0.0071	19.8629		[0.127,0.154]
G	esg_score	0.0301∗∗∗	0.0072	4.1551	0.0000	[0.016,0.044]
S	(Intercept)	0.1207	0.0089	13.5062		[1.103,1.138]
S	esg_score	0.0927∗∗∗	0.0181	5.1151	0.0000	[0.057,0.128]

Notes: ∗p<0.1; ∗∗p<0.05; ∗∗∗p<0.01

In the second period (2019–2021), the intercepts remain large, but some coefficient estimates are more significant, and we do find that larger ESG scores are associated with larger Δ GDP fixed effects, excess changes in GDP *associated* with fixed country characteristics. This holds at the overall ESG level and for both the Social and Governance pillars. Unfortunately, this is not true for the Environmental pillar. We also provide the confidence intervals for the coefficient estimates in the table.

These results are indicative of the positive association of excess ESG scores with excess GDP growth. We acknowledge that there is a range of opinions on this question. Our results are similar to findings in Diaye, Ho, and Oueghlissi (2022), who perform an analysis of OECD countries between 1996 & 2014, and find a positive relationship between ESG and GDP per capita in the long run.

## 5. Applications for ESG investors

In spite of our overall negative (not statistically significant) results, we believe that the notion of demand for ESG factors can be useful for the money management industry. It can be used to tilt allocation towards assets with higher ESG factor exposures that our model believes are in current demand. We consider two ways to exploit country-level demand for ESG: a macro demand flow factors and investor reporting.

### 5.1. Macro ESG demand flow factors

Flow factors have been documented in the literature, in particular for mutual fund flows, for example (Ferson and Kim 2012). Similarly, equity flow factors can be defined at the stock level, and they are based on the hypothesis that flows are persistent and exhibit momentum characteristics. We apply the same principle to ESG factors, which exhibit persistent and consistent demand, using the demand estimates from our model. In short, the idea is to create an allocation that is tilted towards factors that have recently been in high demand according to the model. Technically, this corresponds to factors that have had a positive impact on future returns.

We extend the method of Morgenstern, Coqueret, and Kelly (2021), who introduced the trend following macro ESG efficient frontier. The idea here would consist in tilting the allocation in the direction of those factors which have recently been in demand. This can be achieved with Equation (6) below, which extends the trend strategy weights function (trend signal + ESG tilt) with the demand component:

(6) $${\tilde{w}}_{t}^{\left(i\right)}=\underset{\mathrm{trend}\;\mathrm{signal}}{\underbrace{\sum_{j}\phi \left(\alpha_{t-1}^{i,j}\right)}}+\overset{\mathrm{ESGtilt}}{\overbrace{\kappa \cdot {\tilde{s}}_{t-1}^{\left(i\right)}}}+\underset{\mathrm{demand}\;\mathrm{component}}{\underbrace{\omega \cdot \sum_{l\in Dt}c_{t-1,i}^{\left(l\right)}\cdot d_{t-1}^{\left(l\right)}}},\;$$

The full details of the strategy can be found in Morgenstern, Coqueret, and Kelly (2021). In short, $\alpha_{t-1}^{i,j}$ consists of moving averages at various ranges *j* (from 30 to 360 days) and *ϕ* is a smoothing function. In the second term, ${\tilde{s}}_{t-1}^{\left(i\right)}$ is the sustainability score which is scaled by *κ* which tunes the importance of ESG for the investor. Finally, the last term multiplies the demand estimates $d_{t-1}^{\left(l\right)}$ with ω>0, which determines the relative importance of the flow factor relative to the trend signal and ESG tilt. We provide the time-series of the demand component $\sum_{l\in Dt}c_{t-1,i}^{\left(l\right)}\cdot d_{t-1}^{\left(l\right)}$ in Figure C.16 in the Appendix. The set Dt encompasses the factors for which we wish to use the demand estimates $d_{t-1}^{\left(l\right)}$. This choice may depend on various criteria and we set Dt to be the set of factors which have positive demands.

**Figure 6.**
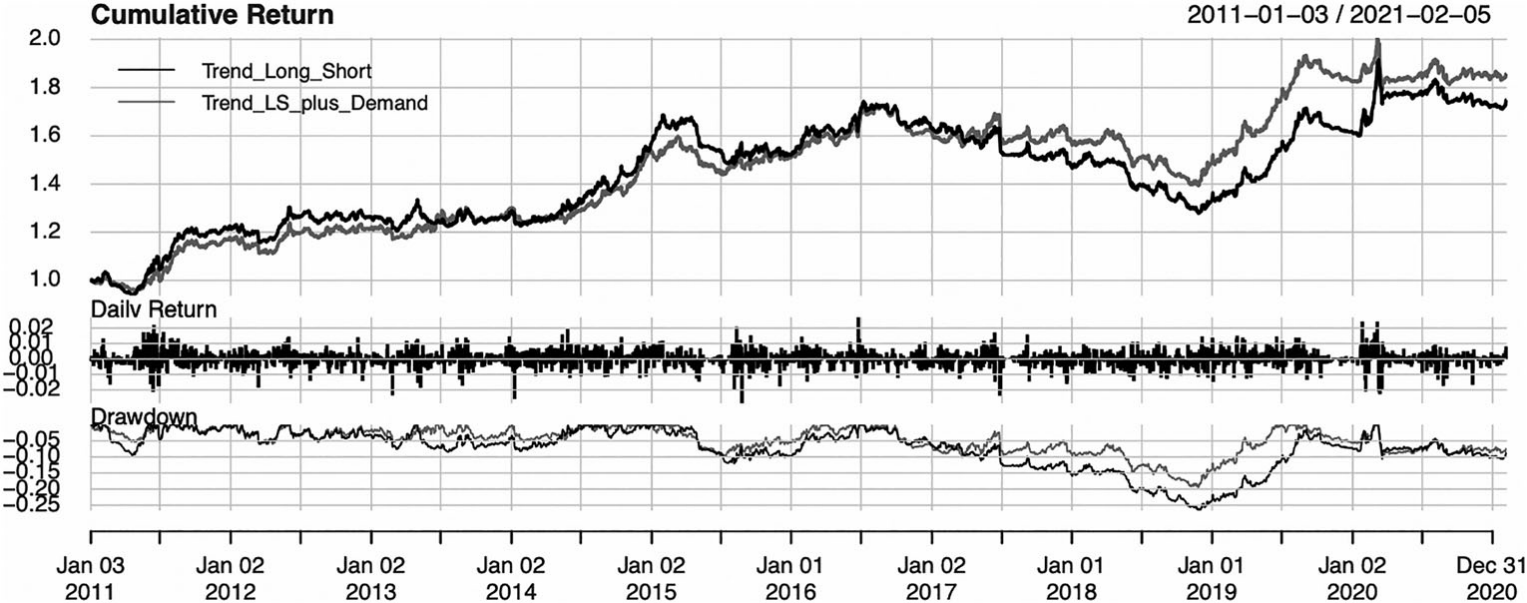
Time-series of cumulative returns. Notes: The strategy is given by Equation (6). Trading starts on 2011-01-03 and ends on 2021-02-05. Trend_Long_Short is the original strategy from Morgenstern, Coqueret, and Kelly (2021) (i.e. with ω=0), while Trend_LS_plus_Demand pertains to the case ω=1/2. The top plot provides cumulative returns, the middle plot provides daily returns and the bottom plot provides the drawdown profiles of the strategies.

We implement the model proposed in Equation (6) for the medium during fixed income trend strategy, with ω=1/2, $Dt=\{l:d_{t}^{\left(l\right)}>0\}$ and with *k* = 0.5. The results are gathered in Figure 6 and Table 2.

**Table 2.** Performance summary.

Strategy	Return	Risk	S.R.	VaR(5%)	Max DD	avg. D. length	T.E.
Trend L/S	5.50%	8.41%	0.65	−0.85%	26.69%	58.05	0.00%
Trend L/S + Demand	6.11%	8.33%	0.73	−0.84%	19.42%	34.61	5.74%

Notes: The strategy is given by Equation (6). Trading starts on 2011-01-03 and ends on 2021-02-05. The first line is the original strategy from Morgenstern, Coqueret, and Kelly (2021) (i.e. with ω=0), while the second one pertains to the case ω=1/2. The metrics are the average return, the volatility (standard deviation of returns), the Sharpe ratio, the Value-at-Risk, the maximum drawdown and the average drawdown length in days, and the tracking error with respect to the first strategy. All values are annualized.

We observe a higher Sharpe Ratio, a lower maximum drawdown and shorter average drawdown length for the strategy by including factor demands relative to the baseline strategy. The cumulative returns show that it is in the bearish periods that the demand-adjusted strategy outperforms, which provides an example of how the demand estimates can be used within an investment strategy.

### 5.2. Investor reporting

An ongoing challenge for asset managers is to incorporate sustainability considerations into their standard reporting to investors, beyond return and risk. In Section 4, we underlined that sustainability metrics at the country level are slowly moving and highly persistent. Hence, most of the variation in ESG exposure in a portfolio comes from changes in the investment strategy. In the context of an ESG-adjusted momentum allocation, such exposures are plotted in Exhibit 9 of Morgenstern, Coqueret, and Kelly (2021).

In this paper, we introduce the demand for ESG factors, which can be used to verify if the composition of the portfolio is aligned with the underlying aggregate demand in ESG factors.

**Figure 7.**
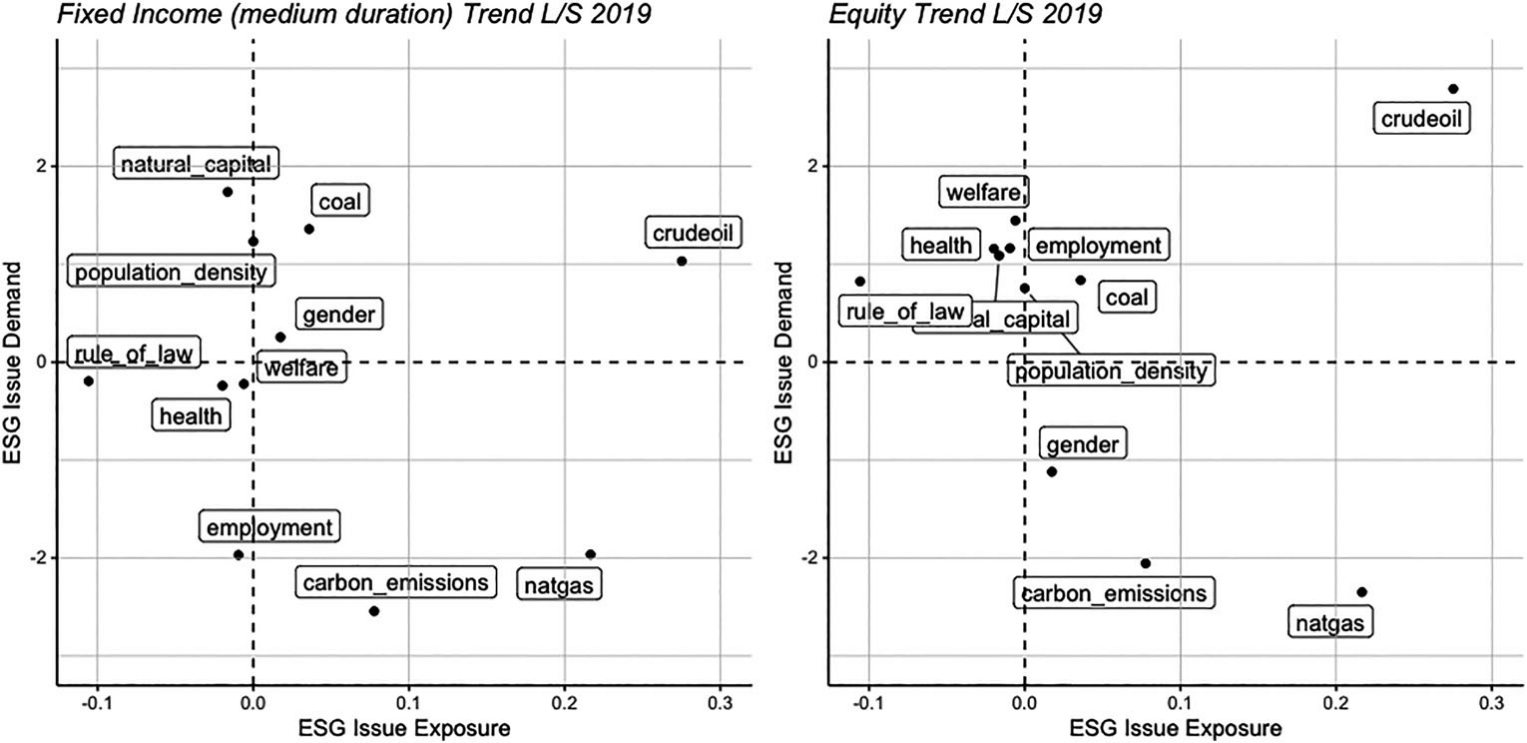
Sustainability demand versus sustainability score. Notes: We locate both items in the plane for fixed income (left panel) and equity (right panel) components of the long-short trend following strategy of Section 5.1. The *y*-axis pertains to standard estimates from the baseline model of Equation (2). The *x*-axis is the weighted score of the strategy (each asset, via its country has a score which is aggregated at the portfolio level via its weight in the portfolio). The snapshot corresponds to December 2019. All components are sorted positively towards sustainability: a high crude oil score means *low* production and consumption of oil.

To this purpose, we consider the trend following strategy from Section 5.1. We compute the exposure (average weighted score) to ESG factors at the issue level and plot this versus the demand for these factors for December 2019 in Figure 7.

We see that natural gas is at the bottom right of each plot. This means that it has a negative demand on markets according to the model, but a large demand adjusted weight in the trend-following strategy. Therefore, the two are not aligned. However, there is a positive alignment with the crude oil factor. We recall that factors are sorted to be positively linked to sustainability; thus, a positive score for crude oil relates to allocations towards countries with low production and consumption of oil. This representation allows for a consistent narrative of how ESG factors, their demand and investment strategies interact.

## 6. Conclusion

In this paper, we seek to measure the sensitivity of broad market indices to ESG scores and their variations. Equivalently, this proxies for the net aggregate demand towards ESG factors at the country level, we do this for both for equity and fixed income markets. We consider different specifications for the estimation of this model, including fixed versus random effects, pillar versus issue levels, several stage estimations and varying window lengths.

Contrasting with the recent sustainability hype, we find no evidence of strong sovereign ESG demand - neither at the pillar nor at the issue level, and across all the models we tested. Nevertheless, we do find that contemporaneously positive ESG scores are associated with excess GDP growth, especially in S and G pillar. Therefore, sovereign ESG can be a useful addition in macro-economic models that seek to explain or forecast aggregate output at national levels.

Our final section provides two examples of how sovereign ESG demand can be used in portfolio management. In particular, we find that macro ESG demand factors can be added to existing macro strategies such as trend following. We concluded by showing how ESG reporting of strategies can be enhanced with macro sustainability demand data.

Our results are built on data from the past 10 to 20 years when sustainability was not yet a priority. With the recent trends that incentivise investors to allocate more and more to sustainable assets, it will be interesting to monitor if our conclusions are altered and if more sustainable markets experience higher or lower returns. If that is not the case, then this would also mean that betting on sustainable countries is not costly, so investors driven by ethical motives can restrict their geographical exposure without having to pay an ESG premium.

## Supplementary Material

Supplemental Material

## Notes

1 Chapter 3, available online at www.esgperspectives.com.

2 The COVID-19 market crash of early 2020 was an event scrutinized by academics with a particular focus on resilient investments. Omura, Roca, and Nakai (2020), Pástor and Vorsatz (2020), Singh (2020) and Xiong (2021) all document an out-performance of sustainable firms during the crisis. A few other papers (e.g. Folger-Laronde et al. 2022; Demers et al. 2021; Mahmoud and Meyer 2021) do not report significant results in this direction.

3 In the sequel, we use ‘*indicator*’, ‘*attribute*’ or ‘*characteristic*’ interchangeably. Characteristics can be standardized into *z*-scores, which is why sometimes the letter *s* is used (for ‘scores’). But henceforth, we will stick with the *c* notation.

4 We collect the data using **R** using (Piburn 2020) for World Bank data, Lahti et al. (2017) for EuroStat data and Grandrud (2020) for IMF data.

5 UN SDGs https://unstats.un.org/sdgs/dataportal.

6 In Morgenstern, Coqueret, and Kelly (2021), the authors used sovereign ESG data to enhance a *tradable* strategy and hence had to use futures data. In Exhibit A1 therein, the universe of future contracts is significantly smaller.

7 For Fixed Income markets, an alternative data provider is S&P. However, except for Africa, S&P provides a narrower geographical coverage and less history. We decided not to mix data sources in order to ensure consistency.

8 We discuss modelling alternatives in Section A in the Appendix.

9 We note a decrease of growth expectation from approximately 3.25% to 2.5% from 2010 to 2020 according to Consensus Economics and the World Bank.

10 We follow the method described in Salibian-Barrera (2005) to calculate the p-values for the robust regression used.
