# Supplementary material for: International market exposure to sovereign ESG
Source: J Sustain Financ Invest. 2022 Nov 25;14(4):968–87. doi: 10.1080/20430795.2022.2148817 (PMC11575848; doi:10.1080/20430795.2022.2148817)
Supplement: Supplemental Material [file TSFI_A_2148817_SM1336.pdf]

International market exposure to sovereign ESG
Supplementary Information

Christian Morgenstern* Guillaume Coqueret† James Kelly‡

November 22, 2022

Appendix A. Discussion on estimation specifications

The theoretical equation $r_{t+1,n} = \alpha_{t+1,n} + \sum_{k=1}^{K} \left(\beta_{t+1}^{(k)} c_{t,n}^{(k)} + \eta_t^{(k)} \Delta c_{t,n}^{(k)}\right) + \epsilon_{t+1,n}$ from Section 3 is a textbook panel (longitudinal) model. We recall that the independent variables $c_{t,n}^{(k)}$ (and changes thereof, $\Delta c_{t,n}^{(k)}$) are the time-t k scores of country n while $r_{t+1,n}$ is the one period ahead return of the asset class in this country. The estimation pertains to the coefficients $\beta_{t+1}^{(k)}$ (changes in scaled demands) and $\eta_n^{(k)}$ (scaled demands).

There are four common types of specifications for the longitudinal model, which we will consider henceforth. We define, $B = I_N \otimes \mathbb{I}_T/T$, which gives the inter-country transformation, and $W = \mathbb{I}_N T - B$, which gives the intra-country transformation. I_N is the $N \times N$ identity matrix, $\mathbb{I}_T$ a vector of length T with all entries equal to 1 and $\otimes$ is the Kronecker product. The specifications are:

- The *fixed effects* model pre-multiplies a standard pooled (OLS) model with the matrix W, as defined above. In this specification averages are removed from the variables and hence there is no intercept term in the regression itself. However, there is a fixed effect (constant) for each country and each estimation period. We will explore below why this is the main model of interest for us.
- The *pooling* model simply ignores the country labels and runs a simple Ordinary Least Squares regression on the raw data. This provides us with estimates at the factor level.
- The *between* model pre-multiplies the standard model with the matrix B, as defined above. This implies a regression of the average covariates and the average response variable.
- The *random effects* model (or error components model) is applicable if the factor estimates are the same for all countries but the intercept terms may be a priori different. In this model specification one can decompose the error term $\epsilon_{t,n}$ further into $\epsilon_{t,n} = \eta_n + \nu_{t,n}$, where η_n is the country specific error term and $\nu_{t,n}$ is the idiosyncratic error term. A time effect μ_t can be introduced as well as twoway effects which include both time and individual effects.

*Imperial College London, MRC Centre for Global Infectious Disease Analysis, Norfolk Place, London W2 1PG, UK. Email c.morgenstern@imperial.ac.uk (corresponding author)

†Associate Professor, EMLYON Business School, 23 avenue Guy de Collongue, 69130 Ecully, FRANCE. E-mail: coqueret@em-lyon.com

‡Portfolio Manager, Connected Asset Management, 27-29 Cursitor Street, London EC4A 1LT, UK. Email: ppybjk@gmail.com

In Figure C.4, in the Appendix, we provide a plot of the estimation for the different models. The Between Model results are generally of limited use as they tell us inter-country estimates and they are small in magnitude. The results of the estimation of the pooling model are broadly in line with estimates of the fixed-effects model, however this estimation does not allow the extraction of the fixed effect values which can be useful, as we see in our section on Estimation.

We use an F-test to establish if effects are present. We observe a range of p-values across the macro sustainability demand models we consider. In particular for models at the issue level we observe p-values below 0.05. In terms of the economic context the pooling model gives us estimates which allow us to measure the demand for sustainability but ignores country labels. The fixed effects are of interest, both the level and the dispersion of them as we will explore in Section 4.5, as they give us a country specific measure.

Random effects models are estimated with Maximum Likelihood (ML) rather than Ordinary Least Squares. With ML estimation, we do not have the finite sample results that allow to use the t-distribution, but we have asymptotic results that allows us to use the standard normal distribution and hence we report z-values rather than t-values for these models. We run the Random effects specification for demand model proposed in Equation (1) and note that the *individual* effects are all zero. This is not unusual and can occur - we refer the reader to Andrews (1999) who covers this effect in more generality. As the individual effects are zero the estimates are the same as for the pooled model specification. We can further more consider the random effects model with time effects and two-way effects. The question if a *fixed effects* or *random effects* model is more appropriate is answered by the Hausman test.

Table A.1 contains the p-values of the Hausman test and we pick the *fixed effects* model specification in all cases except for Equities in the model specification without Macro Variables.

Table A.1: Hausman Test for the Macro Sustainability Demand Model

p-values	Demand Model Eq (1)		Demand Model Eq (2)	
	Equity	Fixed Income	Equity	Fixed Income
Pillar	0.724	0.0189**	0.0167**	0.0005***
Issue	0.0873*	0.0041***	0***	0.0309***
Note:				*p<0.1; **p<0.05; ***p<0.01

Appendix B. Tables

Table B.2: **ESG Pillars, Issues and Sub-Issues.** For the sources, GAR stands for Global Atmospheric Research, BPSR for BP Statistical Review and HF for the Heritage Foundation. The method is understood as follows: L (for low) means that a low value of the indicator is preferable, H (for High) means that high values are favored.

ESG Metric				
Pillar	Issue	Sub_issue	Method	Source
E	carbon_emissions	Carbon Emissions from Fossil Fuel Use (Mn Metric Tonnes)	L	BPSR
E	carbon_emissions	Carbon Emissions from Fossil Fuel Combustion (Mt CO2)	L	GAR
E	carbon_emissions	Carbon Emissions per Capita (tCO2/cap) (Fos. Fuel Combust.)	L	GAR
E	carbon_emissions	Carbon Emissions per GDP (tCO2/kUSD) (Fos. Fuel Combust.)	L	GAR
E	coal	Coal Consumption (Exajoules)	L	BPSR
E	coal	Coal Production (Exajoules)	L	BPSR
E	crudeoil	Crude Oil Consumption (Thousand Barrels per day)	L	BPSR
E	crudeoil	Crude Oil Production (Thousand Barrels per day)	L	BPSR
E	natgas	Estimated Flared Volumes (in BCM)	L	World Bank
E	natgas	Natural Gas Consumption (in exajoules)	L	BPSR
E	natgas	Natural Gas Production (in exajoules)	L	BPSR
E	natural_capital	Adjusted Savings: Natural Resources Depletion (% GNI)	L	World Bank
E	natural_capital	Adjusted Savings: Net Forest Depletion (% GNI))	L	World Bank
G	risk_rating	Property Rights	H	HF
G	risk_rating	Regulation Freedom	H	HF
G	risk_rating	Labor Freedom	H	HF
G	rule_of_law	Rule of Law	H	World Bank
G	rule_of_law	Control of Corruption Index	H	World Bank
G	rule_of_law	Government Effectiveness	H	World Bank
S	employment	Ages 15-24 Employment to Population Ratio	L	World Bank
S	employment	Vulnerable Employment (% of total emp.)	L	World Bank
S	gender	Ratio of Female to Male Labor Force Participation Rate	H	World Bank
S	gender	% of Seats Held by Women in National Parliament	H	World Bank
S	health	Life Expectancy at Birth	H	World Bank
S	health	Infant Mortality Rate (per 1,000 births)	L	World Bank
S	health	Prevalence of Undernourishment (% of pop.)	L	World Bank
S	welfare	GINI Income Inequality	L	World Bank
S	welfare	UN Human Development Index	H	World Bank
S	welfare	Poverty Headcount Ratio (% of pop. at national poverty line)	L	World Bank

List of Countries for Fixed Income markets [Data Source: BBG]
Australia, Austria, Bahrain, Belgium, Bermuda, Brazil, Bulgaria, Canada, Cayman Islands, Chile, China, Colombia, Croatia, Cyprus, Czech Republic, Denmark, Egypt, El Salvador, Estonia, Finland, France, Germany, Greece, Guernsey, Hong Kong, Hungary, Iceland, India, Ireland, Israel, Italy, Japan, Jersey Channel Islands, Kazakhstan, Latvia, Lithuania, Luxembourg, Malaysia, Mexico, Morocco, Netherlands, New Zealand, Norway, Panama, Peru, Philippines, Poland, Portugal, Qatar, Romania, Russia, Saudi Arabia, Singapore, Slovakia, Slovenia, South Africa, South Korea, Spain, Sweden, Switzerland, Taiwan, Thailand, Trinidad and Tobago, Tunisia, United Arab Emirates, United Kingdom, United States, Uruguay, Venezuela

List of Countries for Equity markets [Data Source: MSCI]
Argentina, Australia, Austria, Bahrain, Bangladesh, Belgium, Bosnia and Herzegovina, Botswana, Brazil, Bulgaria, Canada, Chile, China, Colombia, Croatia, Czech Republic, Denmark, Egypt, Estonia, Finland, France, Germany, Ghana, Greece, Hong Kong, Hungary, India, Indonesia, Ireland, Israel, Italy, Ivory.coast, Jamaica, Japan, Jordan, Kazakhstan, Kenya, Korea, Kuwait, Lebanon, Lithuania, Malaysia, Mauritius, Mexico, Morocco, Netherlands, New Zealand, Nigeria, Norway,

Table B.3: **Summary of data sources for Indices.** BBG stands for Bloomberg, S&P for Standard and Poor's and MSCI is Morgan Stanley Capital International. The full list is available upon request.

Asset Class	Data Source	Continent	Count	Earliest Start	Latest Start
Fixed Income	BBG	Africa	4	2000-10-31	2007-08-31
Fixed Income	BBG	Americas	13	2000-10-31	2007-02-28
Fixed Income	BBG	Asia	16	2000-10-31	2006-12-29
Fixed Income	BBG	Europe	29	2000-10-31	2007-07-31
Fixed Income	BBG	Oceania	2	2000-10-31	2000-10-31
Fixed Income	BBG	Other	5	2000-10-31	2006-11-30
Fixed Income	S&P	Africa	13	2008-06-30	2017-02-28
Fixed Income	S&P	Americas	1	1994-01-31	1994-01-31
Fixed Income	S&P	Asia	10	2007-01-31	2015-06-30
Fixed Income	S&P	Europe	19	2000-01-31	2014-01-31
Fixed Income	S&P	Oceania	2	2005-01-31	2014-11-28
Fixed Income	S&P	Other	5	2000-01-31	2020-02-29
Equity	MSCI	Africa	11	1993-01-29	2016-12-30
Equity	MSCI	Americas	9	1970-01-30	2017-06-30
Equity	MSCI	Asia	24	1970-01-30	2014-09-30
Equity	MSCI	Europe	27	1970-01-30	2008-07-31
Equity	MSCI	Oceania	2	1970-01-30	1988-01-29
Equity	MSCI	Other	7	1970-01-30	2016-12-30

Oman, Pakistan, Panama, Peru, Philippines, Poland, Portugal, Qatar, Romania, Russia, Saudi Arabia, Senegal, Serbia, Singapore, Slovenia, South Africa, Spain, Sri Lanka, Sweden, Switzerland, Taiwan, Thailand, Trinidad and Tobago, Tunisia, Turkey, Ukraine, United Arab Emirates, United Kingdom, USA, Vietnam, Zimbabwe

List of Countries for MSCI Developed markets [Data Source: MSCI]
Australia, Austria, Belgium, Canada, Denmark, Finland, France, Germany, Hong Kong, Ireland, Israel, Italy, Japan, Netherlands, New Zealand, Norway, Portugal, Singapore, Spain, Sweden, Switzerland, United Kingdom, United States

List of Countries for MSCI Emerging markets [Data Source: MSCI]
Brazil, Chile, China, Colombia, Czech, Egypt, Greece, Hungary, India, Indonesia, Kuwait, Malaysia, Mexico, Peru, Philippines, Poland, Qatar, Saudi Arabia, South Africa, South Korea, Taiwan, Thailand, Turkey, UAE

List of Countries for EU markets [Data Source: MSCI/BBG]
Austria, Belgium, Bosnia and Herzegovina, Bulgaria, Croatia, Denmark, Estonia, Finland, France, Germany, Greece, Guernsey, Hungary, Iceland, Ireland, Italy, Jersey, Latvia, Lithuania, Luxembourg, Netherlands, Norway, Poland, Portugal, Romania, Russia, Serbia, Slovakia, Slovenia, Spain, Sweden, Switzerland, Ukraine, United Kingdom

Appendix C. Figures

Fig. C.1. **Summary statistics for country-level ESG scores.** Countries are ordered according to their average ESG level.

Description	Min	Q1	Mean	Median	Q3	Max	Boxplot
Sweden	0.51	0.87	0.85	0.90	0.93	0.99	
Singapore	0.60	0.73	0.82	0.87	0.90	0.93	
Hong Kong	0.63	0.74	0.81	0.85	0.88	0.92	
Netherlands	0.21	0.68	0.67	0.71	0.73	0.76	
Australia	0.33	0.59	0.63	0.67	0.73	0.76	
United Kingdom	0.42	0.58	0.63	0.67	0.72	0.77	
Canada	0.38	0.54	0.57	0.59	0.64	0.67	
France	0.33	0.52	0.56	0.59	0.62	0.64	
Germany	0.32	0.56	0.55	0.59	0.62	0.66	
Spain	0.33	0.52	0.53	0.54	0.58	0.60	
Japan	0.23	0.29	0.38	0.41	0.45	0.52	
South Korea	0.29	0.35	0.38	0.39	0.40	0.49	
Italy	0.30	0.34	0.37	0.38	0.39	0.41	
Malaysia	0.21	0.28	0.33	0.34	0.37	0.46	
Thailand	0.10	0.15	0.18	0.18	0.20	0.28	
South Africa	0.09	0.12	0.17	0.15	0.20	0.30	
Brazil	-0.10	-0.04	0.01	0.02	0.05	0.11	
Mexico	-0.15	-0.05	-0.01	0.02	0.04	0.07	
India	-0.36	-0.31	-0.29	-0.27	-0.26	-0.23	
United States	-0.85	-0.70	-0.48	-0.35	-0.30	-0.24	
China	-1.08	-1.00	-0.91	-0.95	-0.80	-0.68	

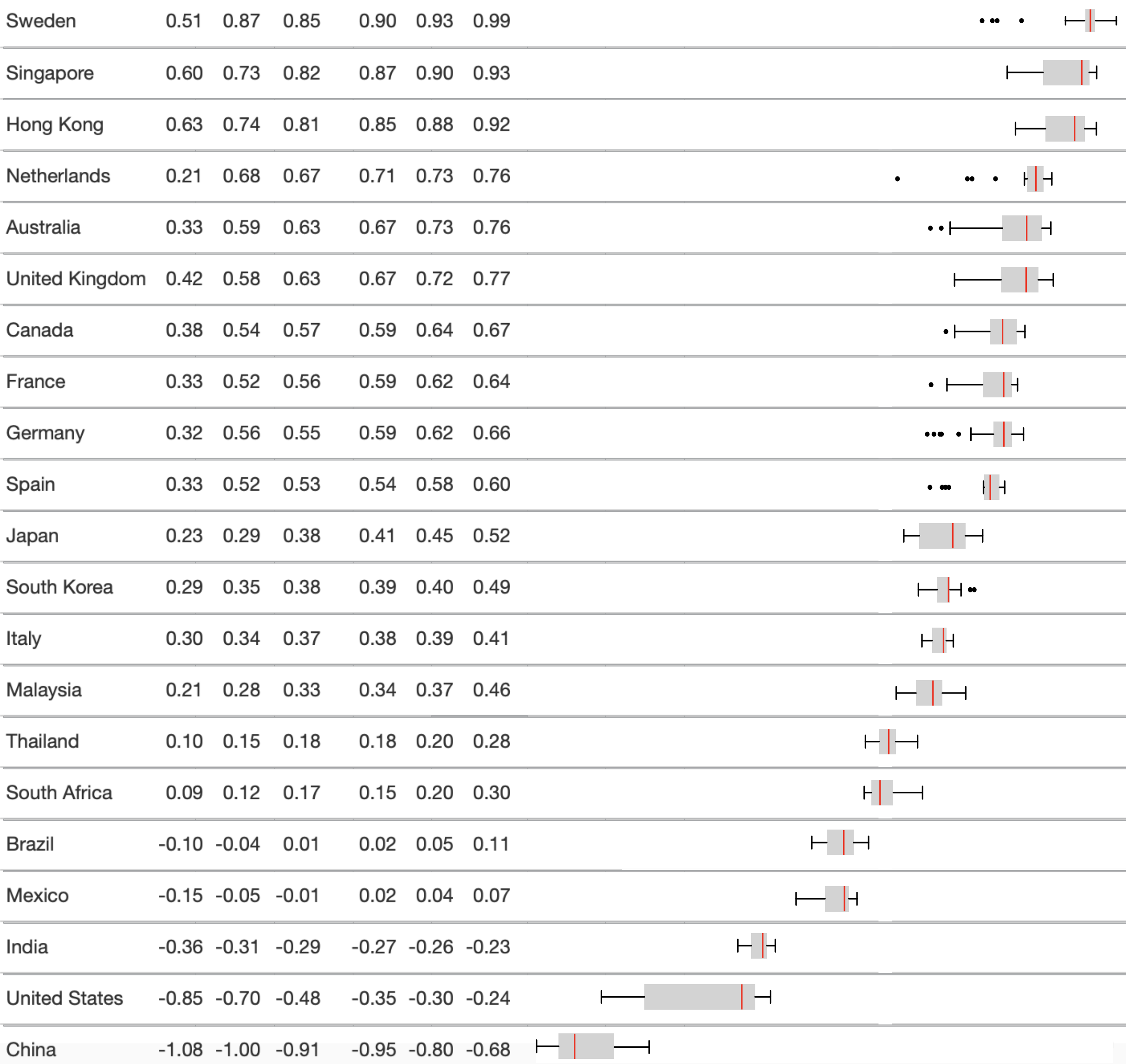

Fig. C.2. **Sub-issue Correlations.** Plot of correlations of sub-issues, including dendogram.

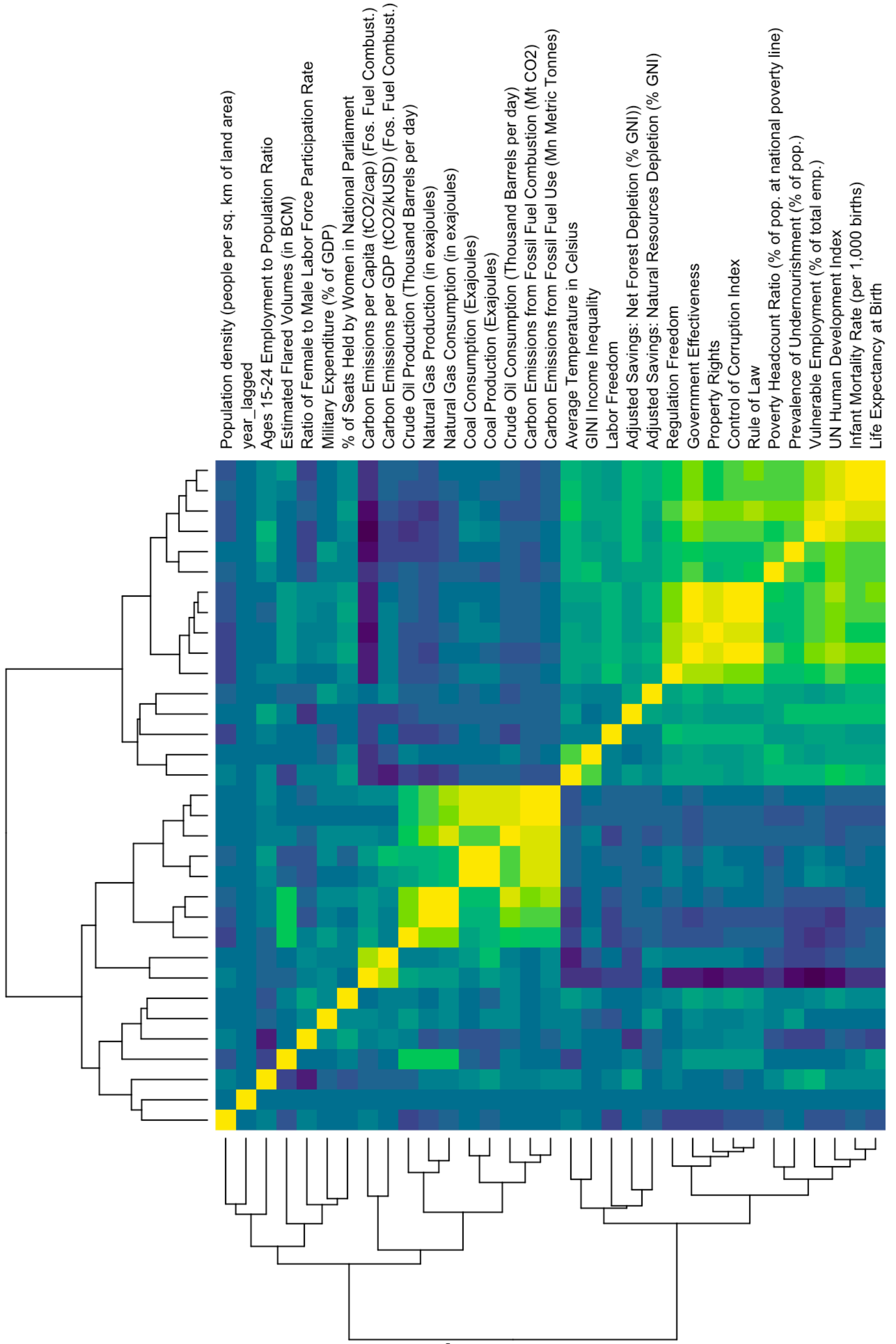

Fig. C.3. **Baseline estimates and macroeconomic controls.** We plot the time-series of t-statistics in fixed-effect panel models (Equation (1)) run on 10 years of data, at the *pillar level*. The panel on the left shows the analysis for fixed income markets while the one on the right pertains to equity markets. The horizontal dashed lines mark the 99% threshold for the significance of the coefficients. The bottom set of plots include macroeconomic controls as defined in model (2).

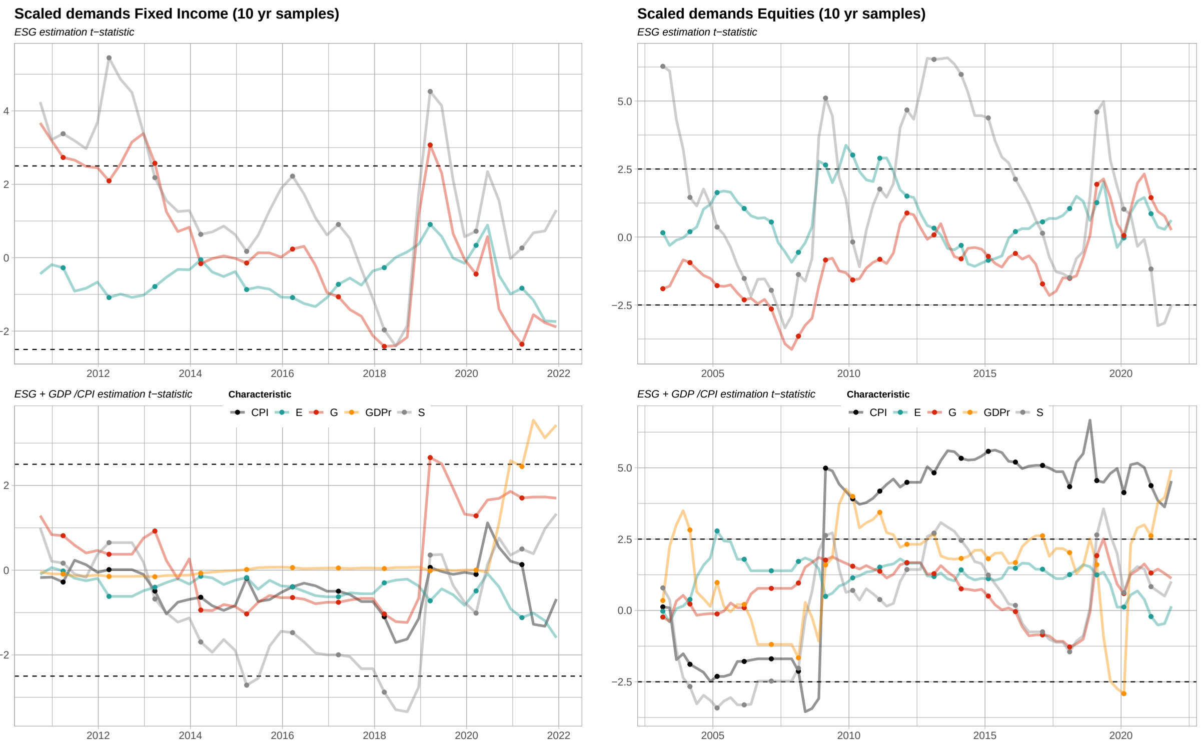

Fig. C.4. **Impact of estimation method for fixed income markets.** We plot the time-series of t-statistics for the panel models (Equation (2)) run on 10 years of data, at the *Pillar level.* the top plot estimates the model with a Fixed Effects specification, the middle plot uses a Between specification and the bottom plot a pooled specification. The horizontal dashed lines mark the 99% threshold for the significance of the coefficients.

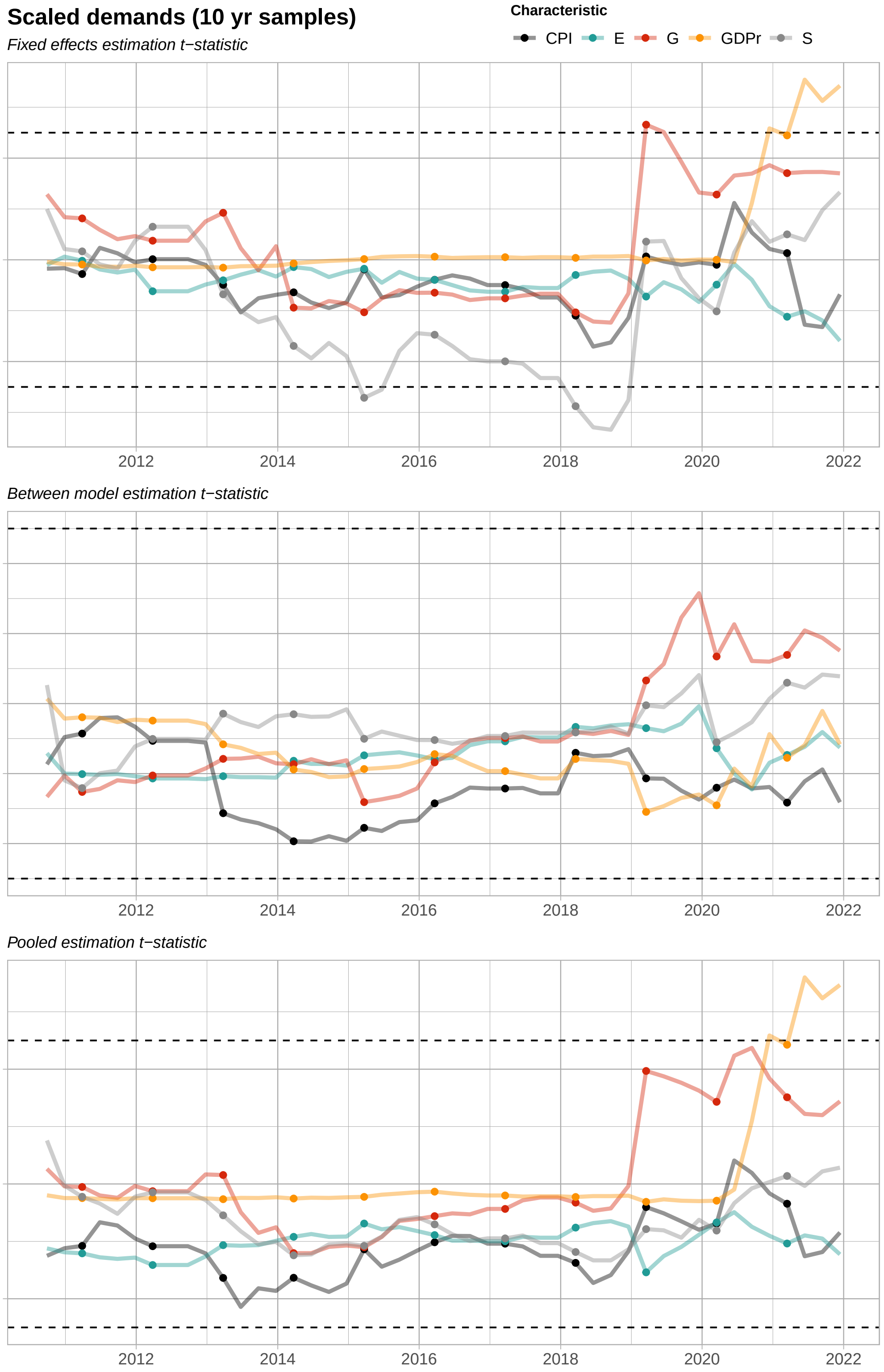

Fig. C.5. **Impact of country selection for fixed income markets.** We plot the time-series of t-statistics for the panel models (Equation (2)) run on 10 years of data, at the *Pillar level*. The estimation is run on a different set of countries as detailed in the sub-plot title. The horizontal dashed lines mark the 99% threshold for the significance of the coefficients.

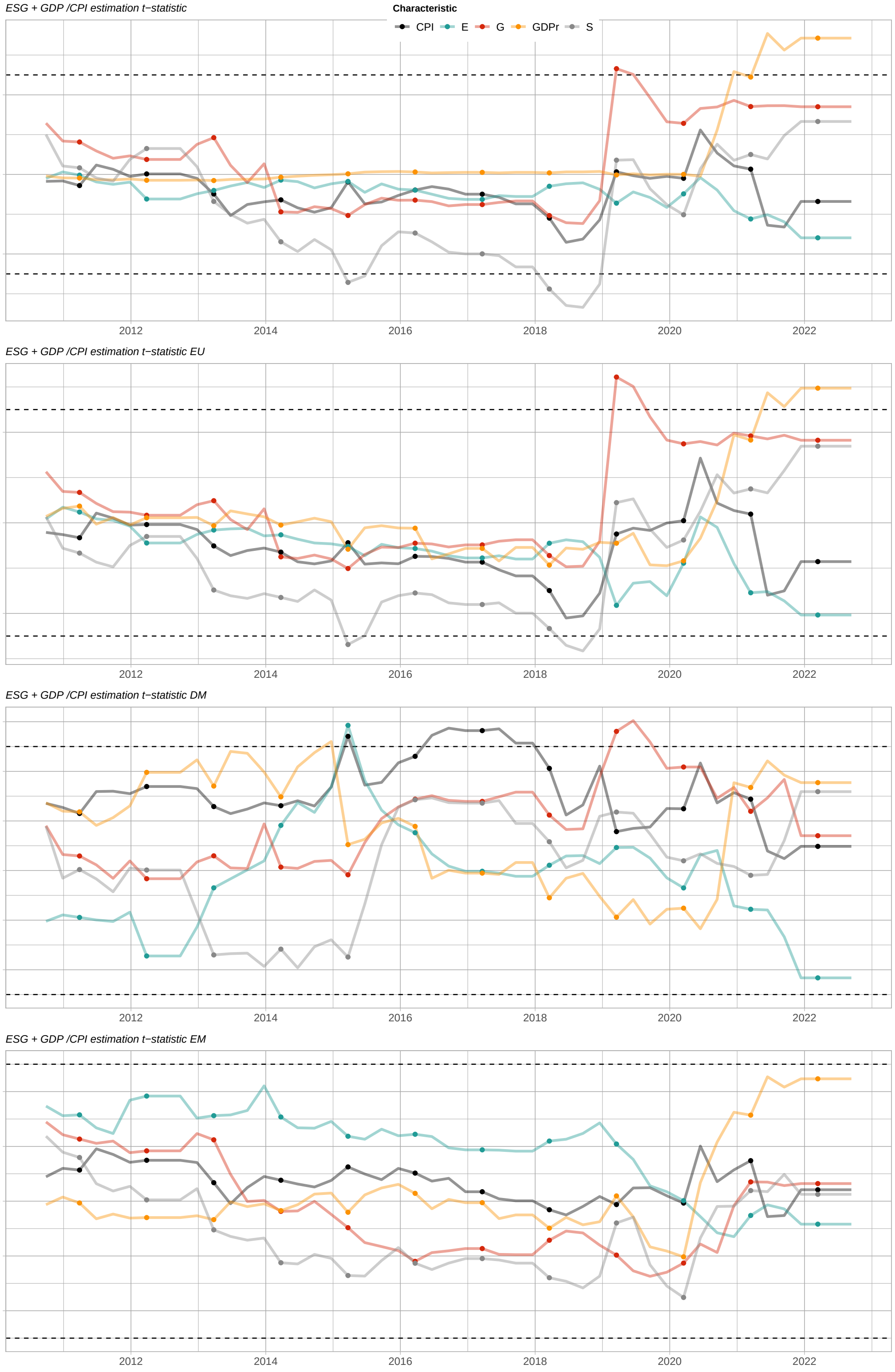

Fig. C.6. **Impact of country selection for equity markets.** We plot the time-series of t-statistics for the panel models (Equation (2)) run on 10 years of data, at the *Pillar level*. The estimation is run on a different set of countries as detailed in the sub-plot title. The horizontal dashed lines mark the 99% threshold for the significance of the coefficients.

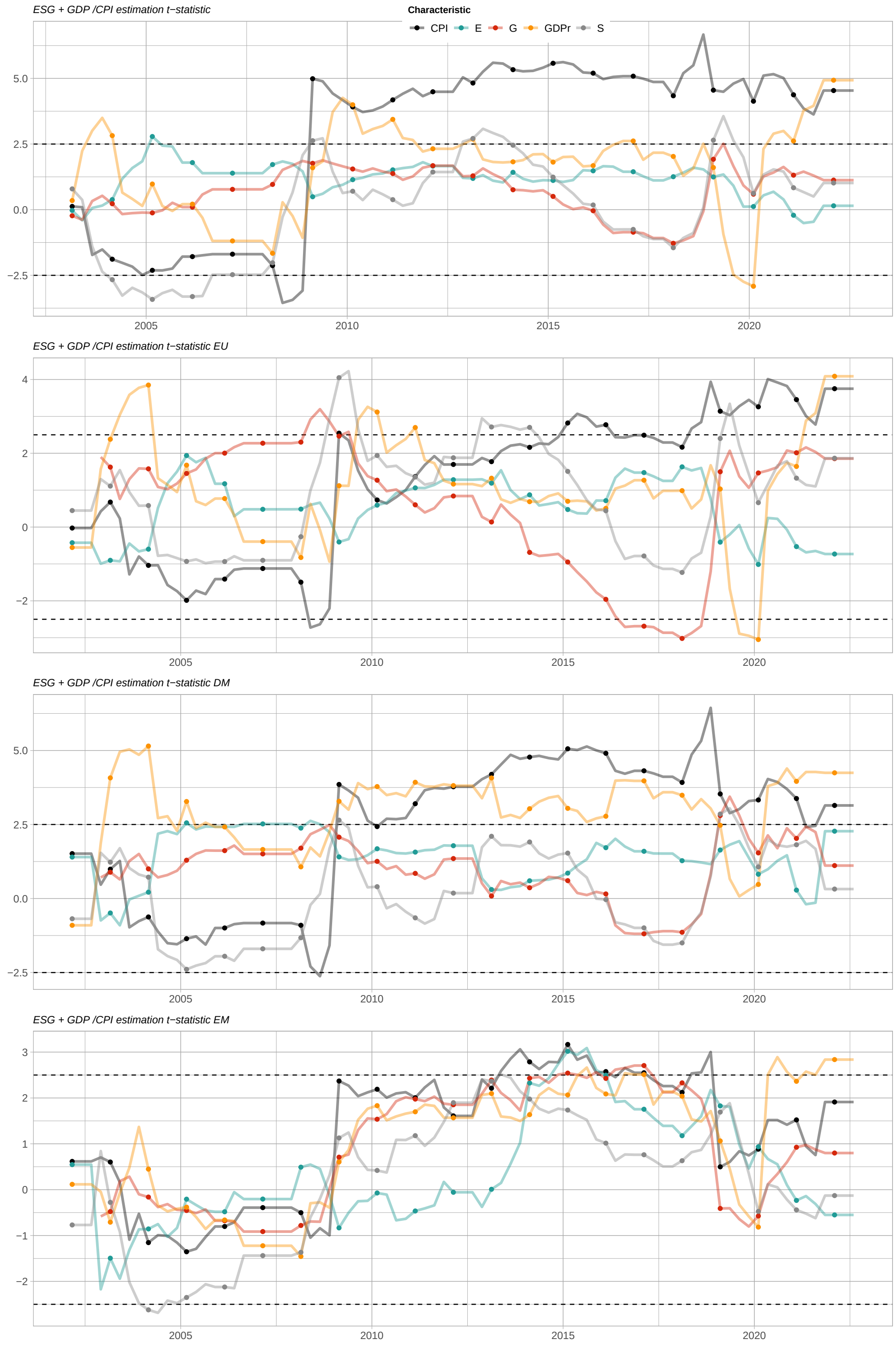

Fig. C.7. **Impact of country selection for equity markets at issue level.** We plot the time-series of t-statistics for the panel models (Equation (2)) run on 10 years of data, at the *Issue level*. The estimation is run on a different set of countries as detailed in the sub-plot title. The horizontal dashed lines mark the 99% threshold for the significance of the coefficients.

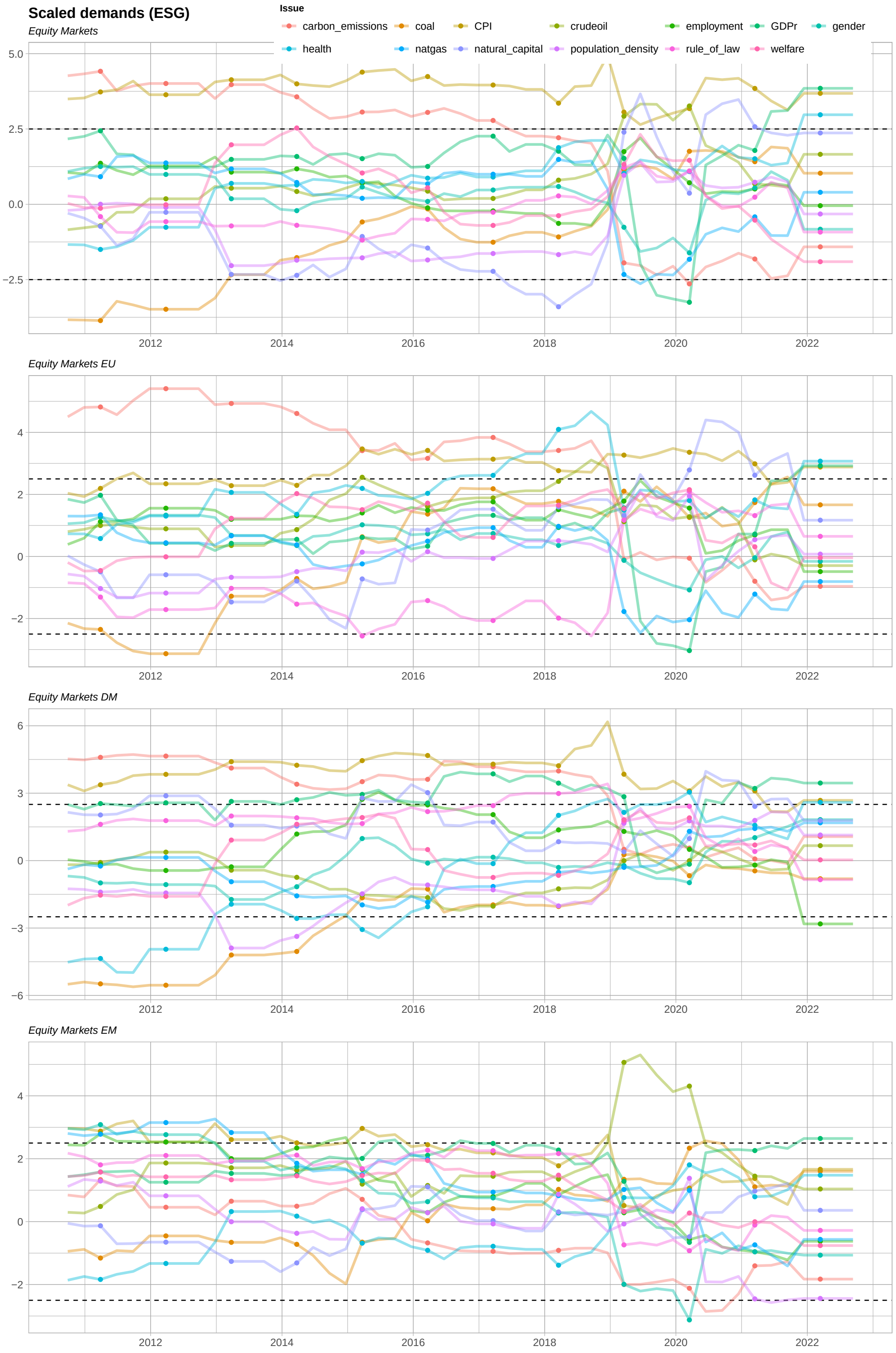

Fig. C.8. **One stage versus two stage model for equity markets.** We plot the time-series of t-statistics in fixed-effect panel models (Equation (2)) run on 10 years of data, at the *Pillar level*, in the top plot. The bottom plot pertains to the two stage model as defined in Equations (3) and (4). The horizontal dashed lines mark the 99% threshold for the significance of the coefficients.

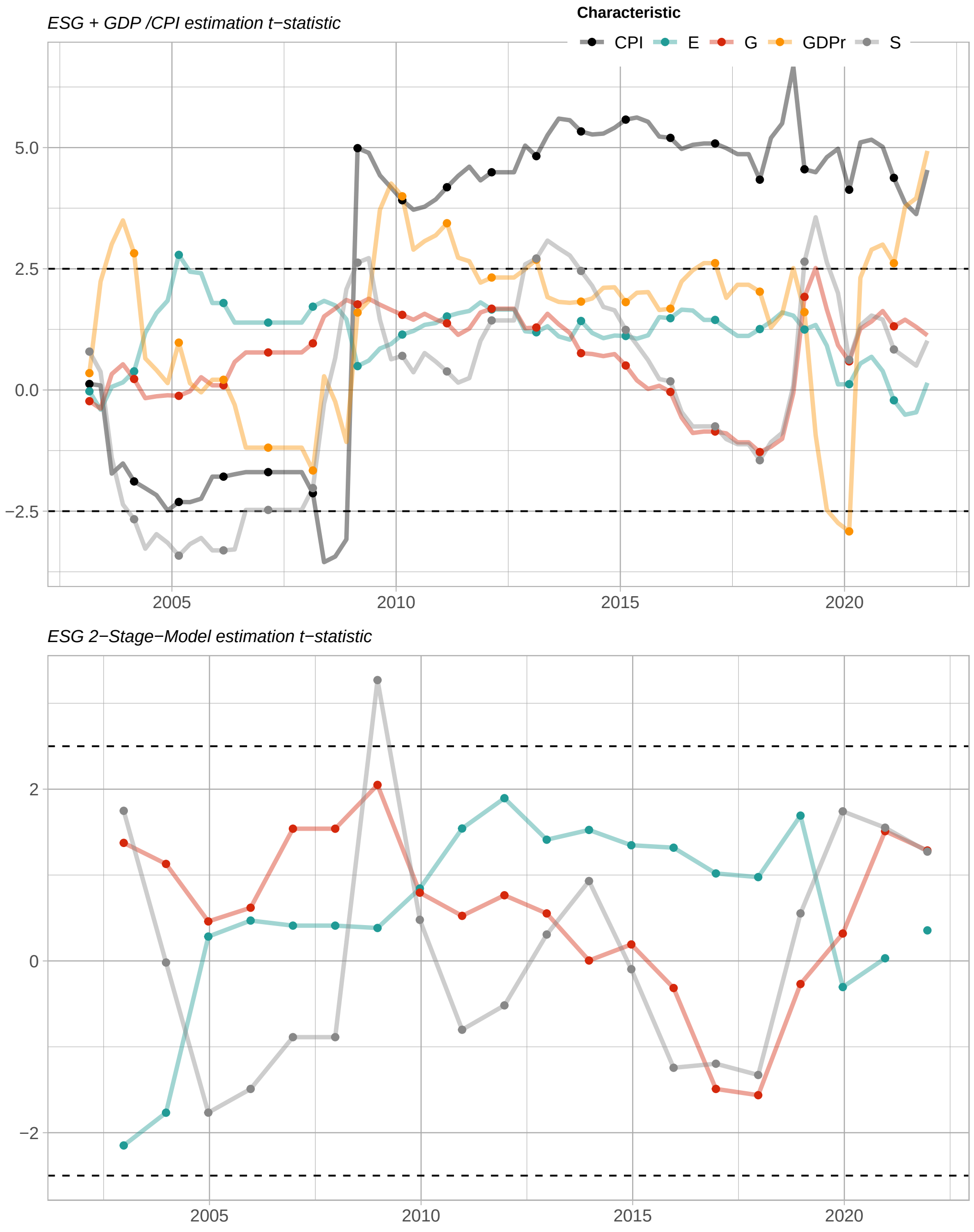

Fig. C.9. **Impact of sample length for equity markets.** We plot the time-series of t-statistics in fixed-effect panel models (Equation (2)), at the *Pillar level*, for equity markets. The top plot is using 5 years of data, the middle plot 10 years of data and the bottom plot 20 years of data. The horizontal dashed lines mark the 99% threshold for the significance of the coefficients.

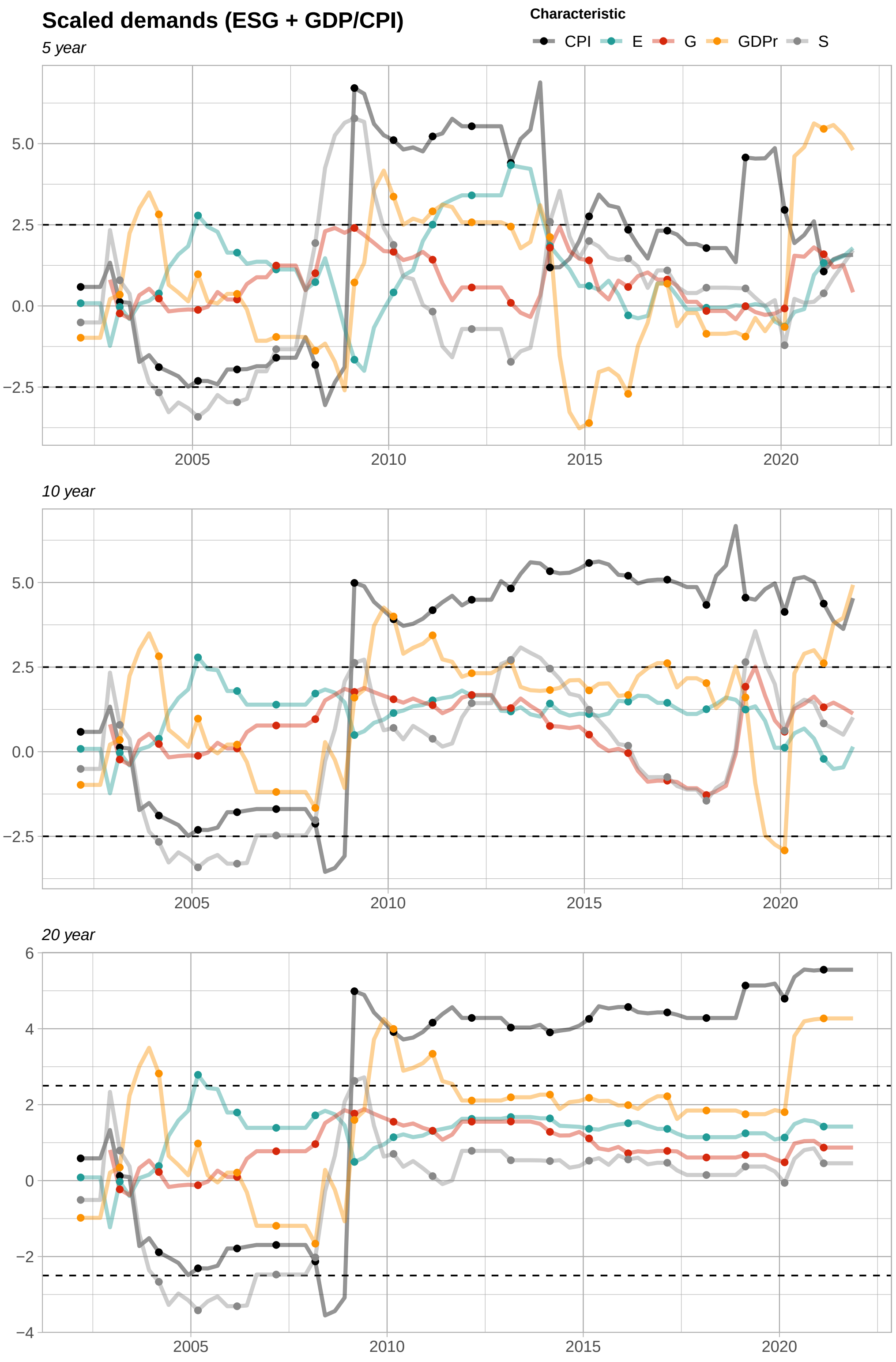

Fig. C.10. **Analysis at issue level for equity & fixed income markets with macro variables.** We plot the time-series of t-statistics in fixed-effect panel models (Equation (2)) estimated on 10 years of data, at the *issue level*. The panel on the top shows the analysis for equity markets while the one on the bottom pertains to fixed income markets. The horizontal dashed lines mark the 99% threshold for the significance of the coefficients.

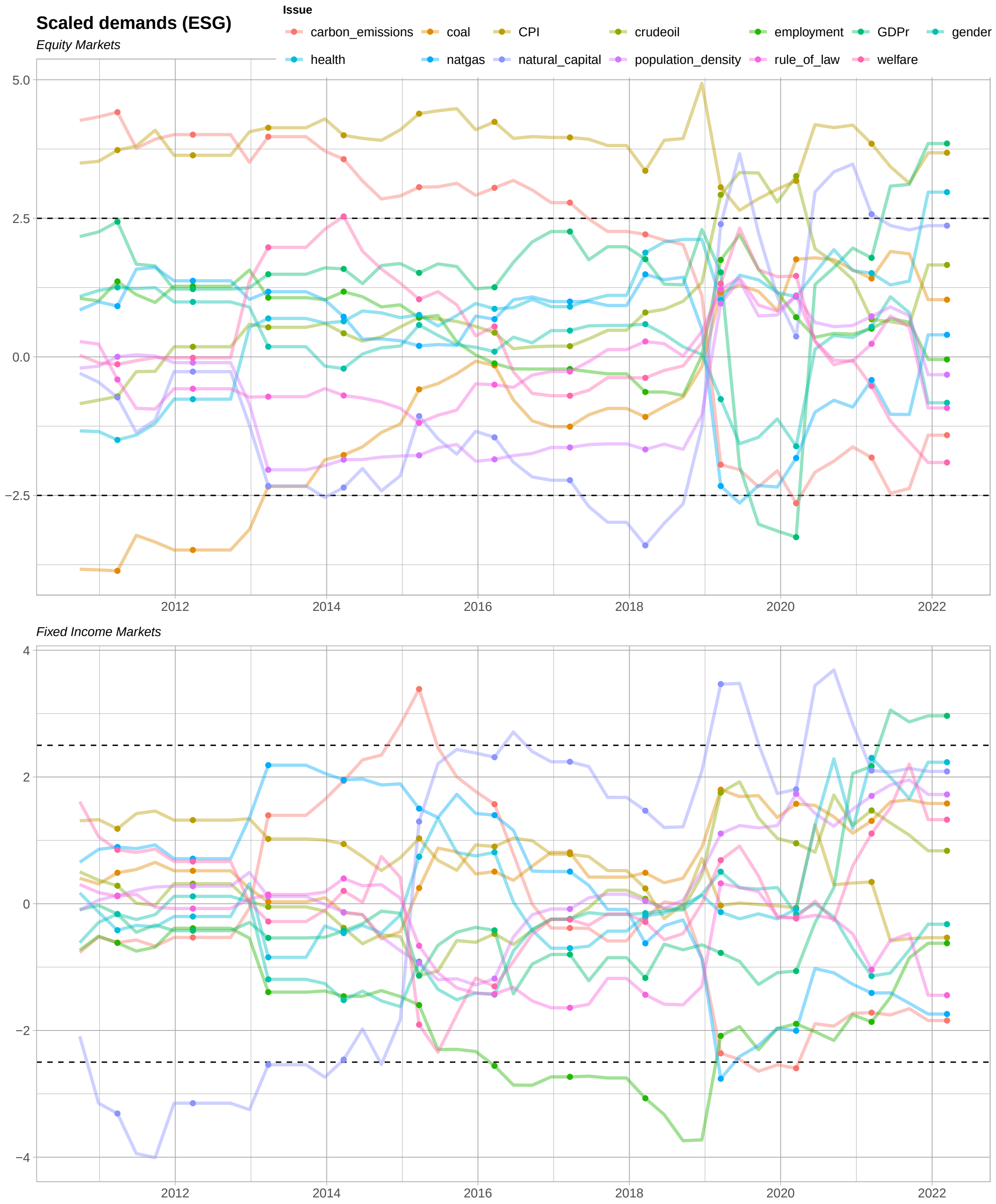

Fig. C.11. **Analysis at issue level for equity & fixed income markets without macro variables.** We plot the time-series of t-statistics in fixed-effect panel models (Equation (1)) run on 10 years of data, at the *Issues level*. The panel on the top shows the analysis for equity markets while the one on the bottom pertains to fixed income markets. The horizontal dashed lines mark the 99% threshold for the significance of the coefficients.

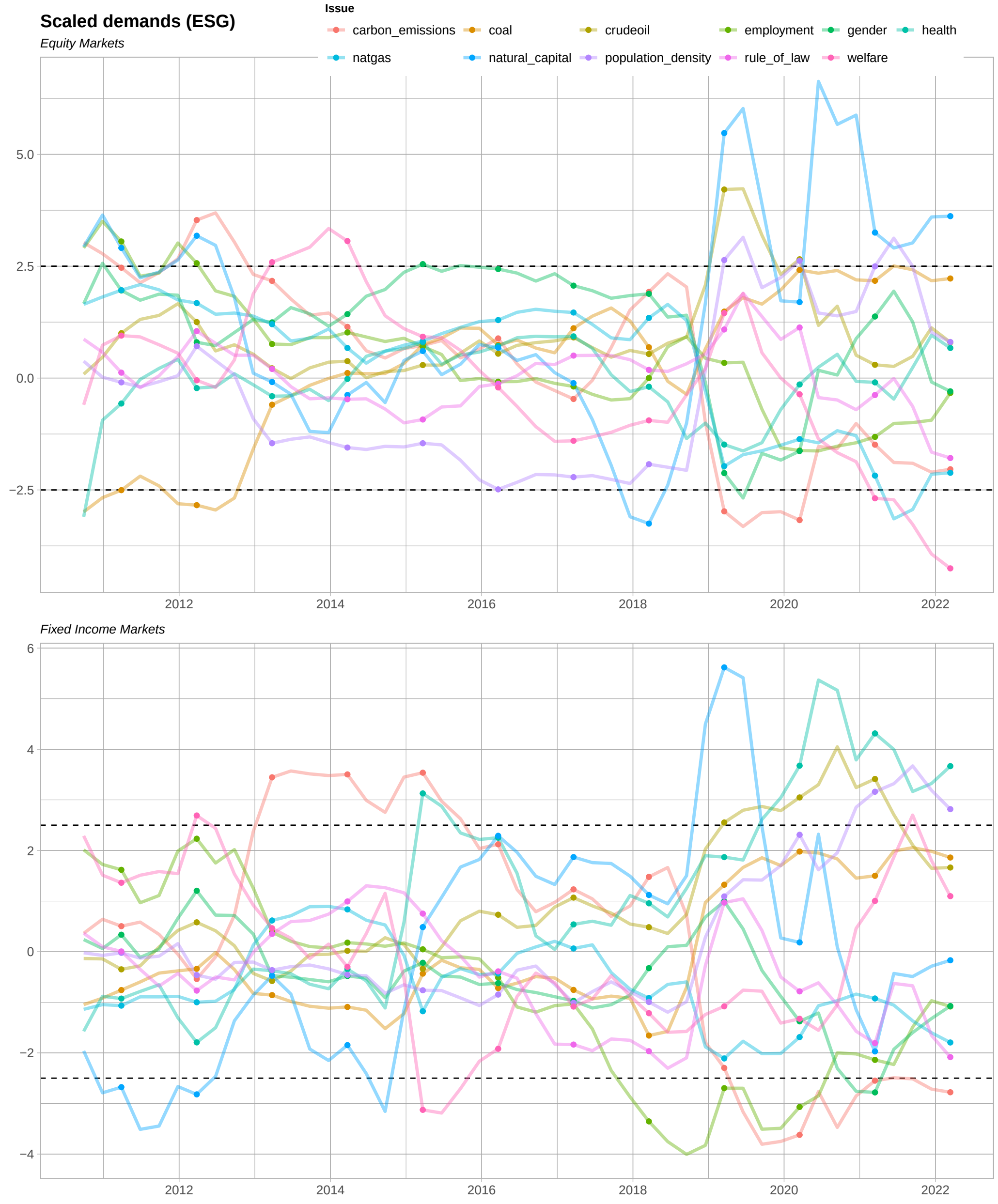

Fig. C.12. **Fixed effects distribution & R^2 for equity & fixed income markets without macro variables.** We plot the quantiles of the fixed effects in fixed-effect panel models (Equation (1)) run on 10 years of data, at the *Pillar level*. The left hand top plots pertains to fixed income markets, the left hand bottom plot pertains to equity markets and the right hand plot displays the R^2 of the models over time.

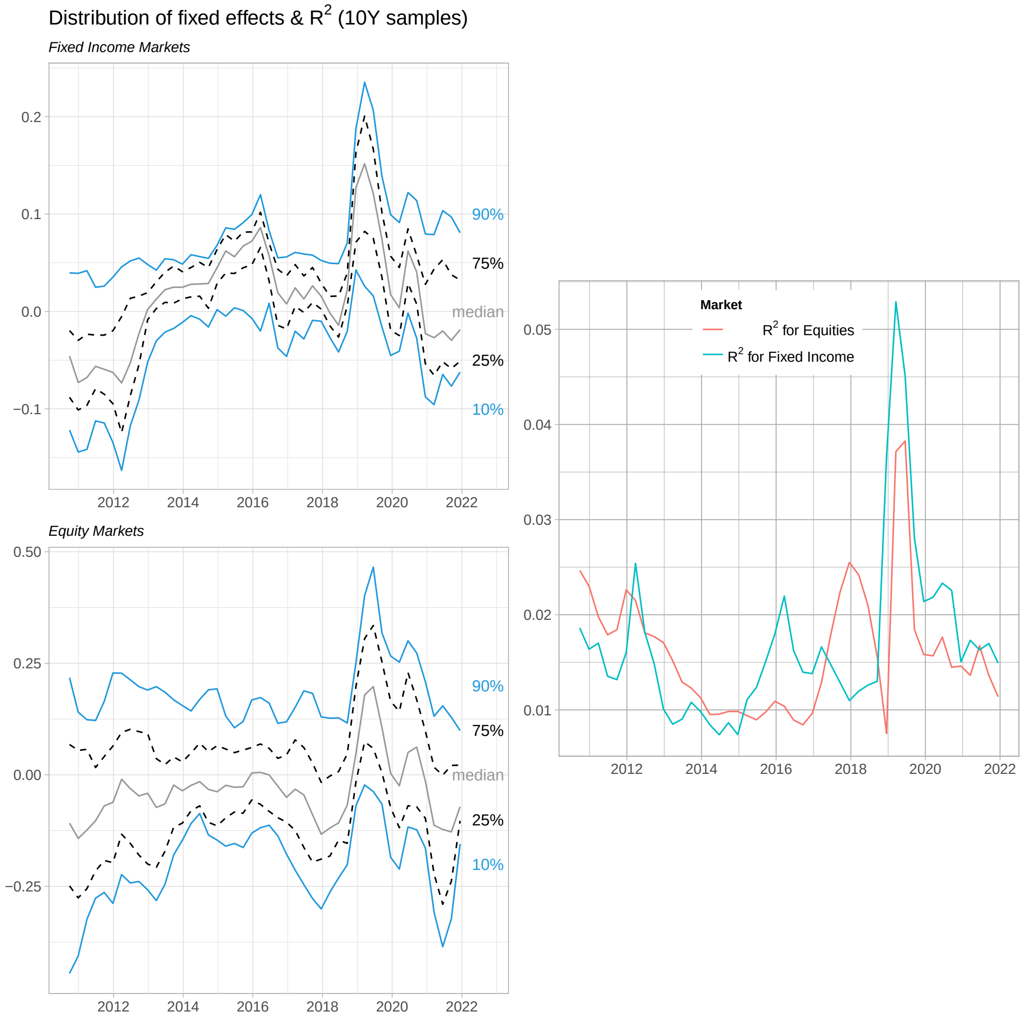

Fig. C.13. **GDP & CPI as explanatory variables only for equity markets.** In the upper panel, We plot the dynamic estimates obtained from model (3) with fixed effects, run on 10 years of data for equity markets. In the lower panel, we show the corresponding quantiles of fixed effects (left) and the R^2 (right).

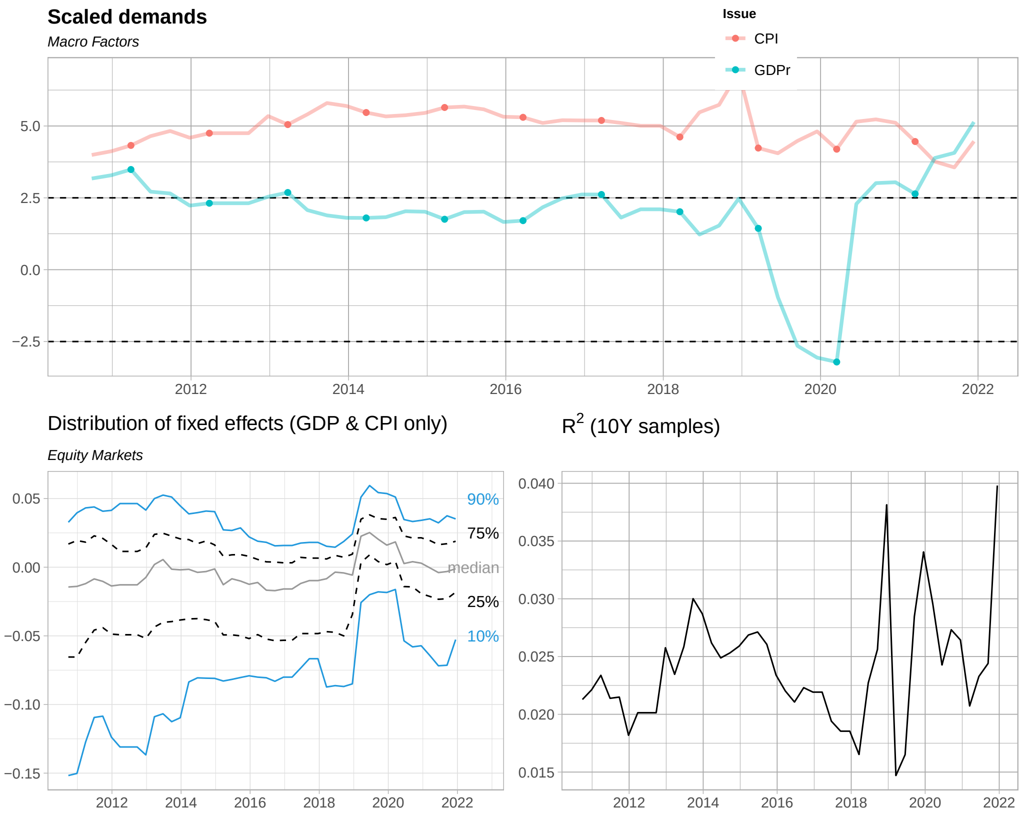

Fig. C.14. **Changes GDP as dependent variable**. In the upper panel, We plot the dynamic estimates obtained from model (5) with fixed effects. In the lower panel, we show the corresponding quantiles of fixed effects (left) and the R^2 (right).

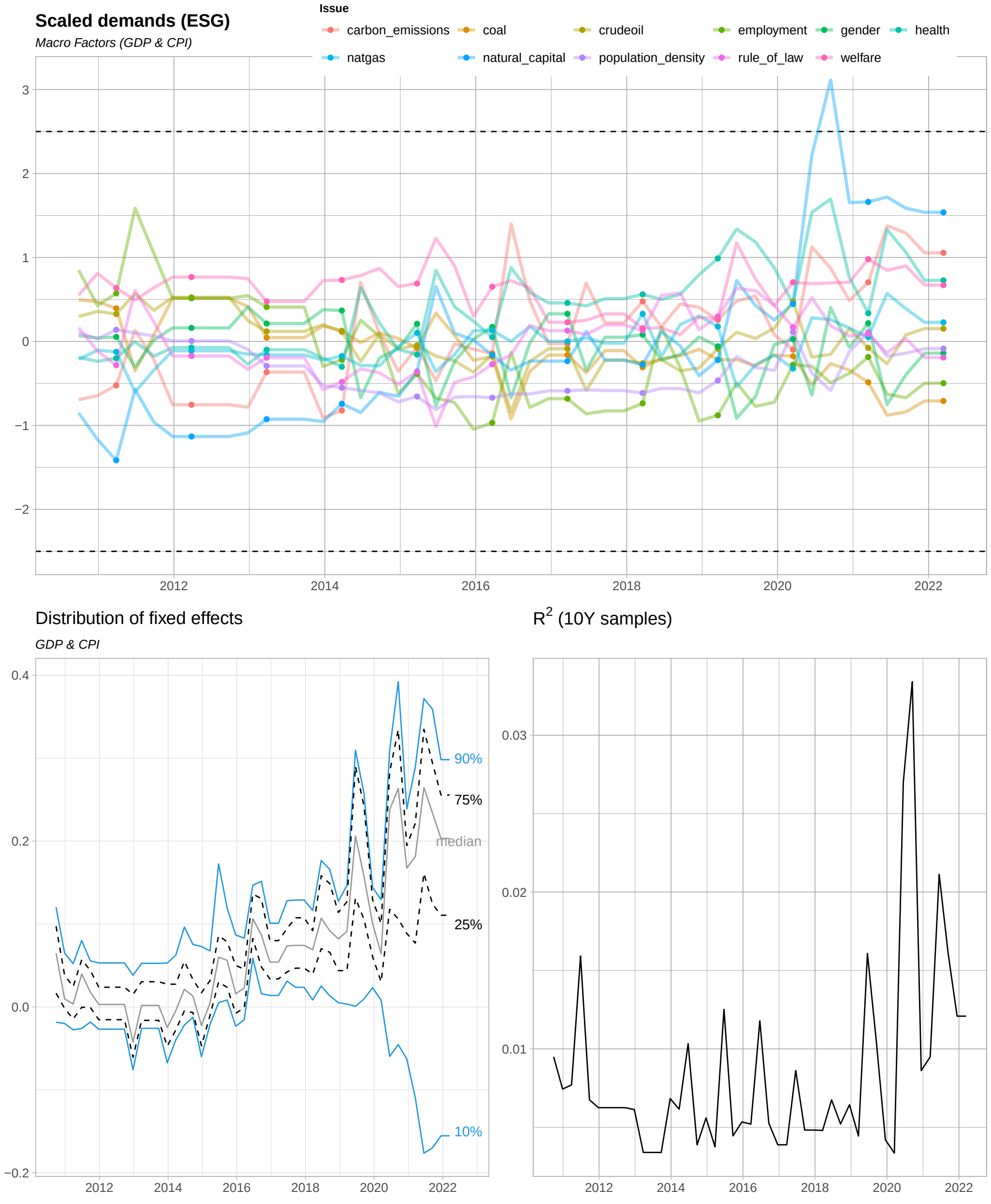

Fig. C.15. **Fixed effects of GDP changes vs ESG issues model.** We plot the fixed effects (y-axis) of model (5), run on 10 years of data, as a function of sustainability scores. The left hand column covers the sub-period 2010-18 and the right hand column the sub-period 2019-21. From top to bottom the plots are for the ESG, E, S and G levels respectively.

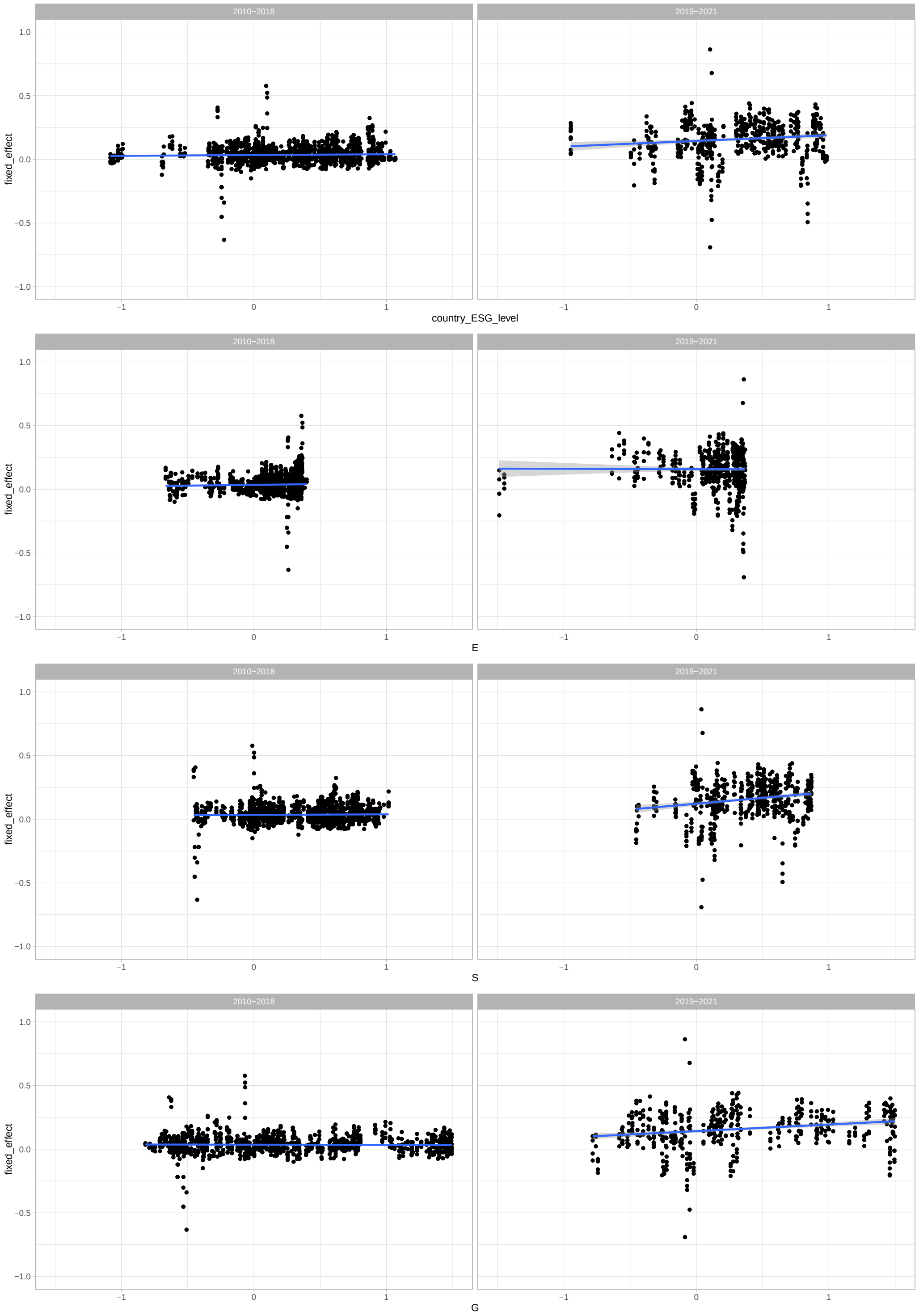

Fig. C.16. **Demand factor for fixed income trend markets.** Time series of the demand factor as defined in Equation (6) for Fixed Income markets from 2011 to 2020.

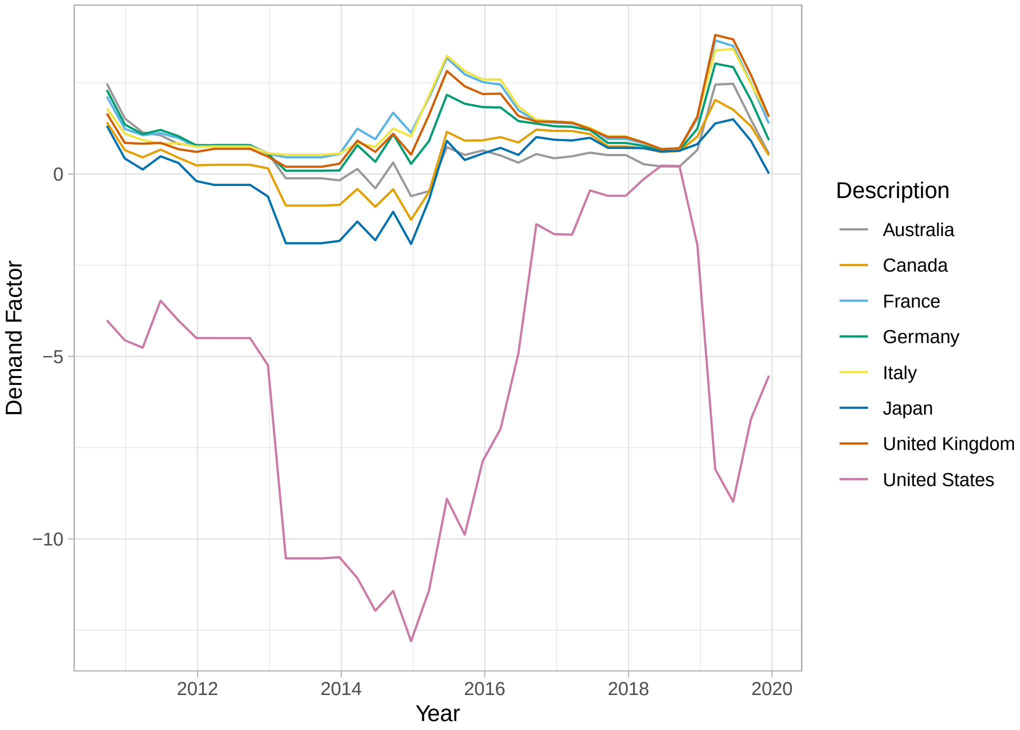

References

Andrews, D. W. K. (1999). Estimation when a parameter is on a boundary. *Econometrica 67*(6), 1341–1383.
